# Availability of the Molecular Switch XylR Controls Phenotypic Heterogeneity and Lag Duration during Escherichia coli Adaptation from Glucose to Xylose

**DOI:** 10.1128/mBio.02938-20

**Published:** 2020-12-22

**Authors:** Manon Barthe, Josué Tchouanti, Pedro Henrique Gomes, Carine Bideaux, Delphine Lestrade, Carl Graham, Jean-Philippe Steyer, Sylvie Meleard, Jérôme Harmand, Nathalie Gorret, Muriel Cocaign-Bousquet, Brice Enjalbert

**Affiliations:** a TBI, Université de Toulouse, CNRS, INRAE, INSA, Toulouse, France; b CMAP, CNRS, Ecole Polytechnique, IP Paris, Palaiseau, France; c TWB, Université de Toulouse, CNRS, INRA, INSA, Ramonville-Saint-Agne, France; d Inria‡; e INRAE, Université de Montpellier, LBE, Narbonne, France; f Institut Universitaire de France‡; Novo Nordisk Foundation Center for Biosustainability; Korea Advanced Institute of Science and Technology

**Keywords:** adaptation, *Escherichia coli*, subpopulations, heterogeneity, metabolic transition

## Abstract

The glucose-xylose metabolic transition is of growing interest as a model to explore cellular adaption since these molecules are the main substrates resulting from the deconstruction of lignocellulosic biomass. Here, we investigated the role of the XylR transcription factor in the length of the lag phases when the bacterium Escherichia coli needs to adapt from glucose- to xylose-based growth. First, a variety of lag times were observed when different strains of E. coli were switched from glucose to xylose. These lag times were shown to be controlled by XylR availability in the cells with no further effect on the growth rate on xylose. XylR titration provoked long lag times demonstrated to result from phenotypic heterogeneity during the switch from glucose to xylose, with a subpopulation unable to resume exponential growth, whereas the other subpopulation grew exponentially on xylose. A stochastic model was then constructed based on the assumption that XylR availability influences the probability of individual cells to switch to xylose growth. The model was used to understand how XylR behaves as a molecular switch determining the bistability set-up. This work shows that the length of lag phases in E. coli is controllable and reinforces the role of stochastic mechanism in cellular adaptation, paving the way for new strategies for the better use of sustainable carbon sources in bioeconomy.

## INTRODUCTION

Microorganisms live in a competitive and fluctuating environment with frequent changes in the nature and availability of nutrients, thus requiring efficient adaptation if they are to continue to grow and survive. Such changes trigger regrowth lag phases defined as the time needed to resume growth either after starvation when a substrate again becomes available (hereby named regrowth lag) or when the cells switch from one substrate to another (hereby named diauxic lag) (1–4). The latter occurs when bacteria are faced with mixed carbon sources. In such a case, they generally use the carbon sources sequentially, starting from their preferred source (1, 5, 6). Use of the secondary substrate is repressed by the preferred one via a mechanism called carbon catabolite repression (7). More particularly, when Escherichia coli cells are cultivated on glucose plus another substrate, their growth is described as diauxic, with exponential growth phases on each substrate interrupted by a diauxic lag phase of minimum or no growth, as first described by Jacques Monod (1). Sequential use is widely considered an adaptation to rapidly consume the most beneficial substrate. This is supported by the fact that carbon sources allowing the higher growth rates are the first to be consumed (5).

Unravelling the mechanisms behind metabolic adaptation is a long-term effort of the microbiology community. For decades, the lag phases before resuming growth have been considered an inevitable temporal constraint to produce the machinery required for growth on this substrate (1, 8, 9). The recent emergence of single cell technologies has undermined this assumption, as it has revealed differential behaviors in cells belonging to the same population when they switch from one substrate to another (10–14). Not all the cells are subject to growth lag: bimodality could be observed, with a fraction of the population not able to grow on the new substrate. This was proposed as a possible explanation for lag phases. However, the mechanisms causing microbial populations to end up in metabolically different phenotypes remain mostly unknown (15–17). The molecular explanations are so far very different from one study to another (17). A stringent response, cell-dependent variations of intracellular contents, low-concentration transcription factors, feedback regulatory circuits, catabolite repression, and epigenetic-related regulatory mechanisms have been reported to play a role so far, depending on the microorganism and conditions (12, 13, 16, 18–24). Due to this profusion of explanations, laying the foundations for metabolic adaptation will require further studies to better understand lag phases and adaptation mechanisms.

The glucose-to-xylose transition is a highly interesting model to explore lag phases in E. coli. This transition is of economic interest, since glucose and xylose are the two main compounds released during degradation of lignocellulose (one of the main renewable alternatives to the use of petrol or gasoline) (25–28). Xylose metabolism is simple, comprises only a few metabolic steps, and is well described, but the mechanisms involved in the switch from glucose to xylose remain to be elucidated. Xylose uptake is mediated through an ABC transporter (encoded by *xylFGH*) or a symporter (XylE) (29, 30). Once in the cytoplasm, xylose is converted into xylulose by the xylose isomerase XylA (31, 32) and phosphorylated into xylulose-5-phosphate by the xylulokinase XylB (33) before entering the pentose phosphate pathway. Xylose metabolism involves two operons (catabolism operon *xylAB* and transporter/regulatory operon *xylFGHR*) that share the same promoter region and are both positively controlled by the XylR transcription factor. When E. coli cells are grown in batches on a mixture of glucose and xylose, they use glucose first and catabolite repression prevents the expression of the two *xyl* operons (34). Consequently, during growth on glucose, the concentration of XylR can be as low as one molecule per cell (35). Once the glucose is exhausted, an increase in the concentration of intracellular cyclic AMP (cAMP) leads to the formation of the cAMP receptor protein (CRP)-cAMP complex, thereby alleviating the glucose-mediated carbon catabolite repression of the promoters (7, 36, 37). Xylose then binds to and activates the transcription factor XylR, which conjointly with the CRP-cAMP complex, triggers the transcription of the *xylAB* and *xylFGHR* operons (34, 38). The binding of XylR-xylose to its own promoter triggers a positive loop that in turn, amplifies its own concentration up to 57 molecules per cell (35). This triggers the production of the enzymatic machinery required for xylose consumption (34). Interestingly, a bimodal induction response to xylose was reported for the *xylAB* promoter with an all or nothing expression (39).

In this study, we used both experimental and modeling approaches to investigate the role of XylR as a molecular switch, defined here as a molecule able to orientate the cellular metabolic fate in an “on/off” manner. We show that XylR promotes the cell’s capacity to resume growth on xylose after the glucose is exhausted. XylR availability controls the length of the lag and influences the emergence of a subpopulation able to resume exponential growth on xylose. This work highlights the role of molecular switches in the establishment of phenotypic heterogeneity and proposes the glucose-xylose transition as a paradigm to understand lag phases and metabolic adaption.

## RESULTS

### The lag time required to adapt to xylose depends on the genetic background of E. coli.

**FIG 1:**
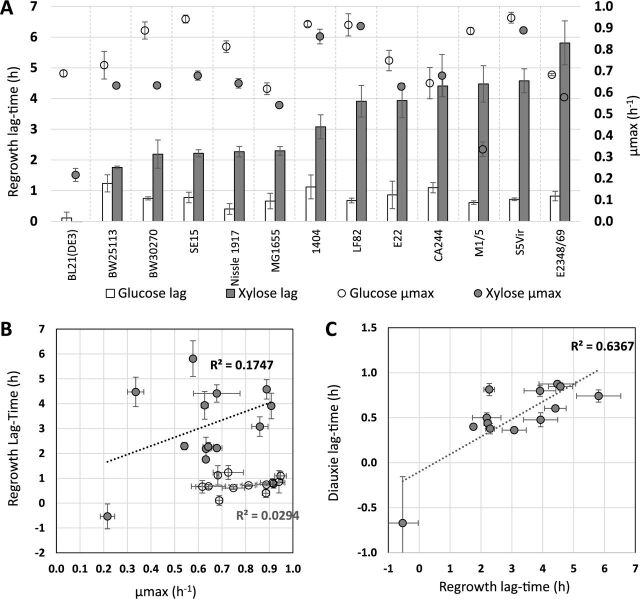
The length of the glucose-xylose lags in E. coli is background dependent. Thirteen different strains of E. coli were cultivated by switching strains previously grown on M9 glucose medium to 96-well microplates filled with 200 μl of fresh M9 xylose or M9 glucose medium. (A) Lag times (bars) and maximum growth rates (μmax) (circles) after the switch to glucose (white) or xylose (gray) for the 13 E. coli strains (*n* = 9 with 3 technical replicates for each of the 3 biological replicates). (B) Scatterplot of the regrowth lag time on xylose or glucose as a function of the maximum growth rate on xylose or glucose for each strain (gray circles for xylose, white circles for glucose). (C) Scatterplot of glucose-xylose diauxic lag times versus regrowth lag times (cells grown overnight on glucose switched to xylose). For diauxic growth, strains previously grown on glucose were transferred to a glucose-xylose M9 medium (12.5% glucose and 87.5% xylose). Diauxic lags were calculated as explained in Materials and Methods. The negative regrowth lag time calculated for the BL21 strain was due to a higher growth rate when this strain resumed growth on xylose than its maximum growth rate during its exponential growth on xylose. For diauxic lags, *n* = 9 for strains BW25113, 1404, LF82, and S5Vir, and *n* = 6 for strains MG1655, E2348/69, E22, BL21(DE3), BW3070, CA244, M1/5, Nissle 1917, and SP15 strains.

To check the consistency of the glucose-xylose transition, we first explored the variability of the lag duration in 13 E. coli strains selected for their origin, lifestyle, and phylogenetic group (Table 1). We quantified their capacity to resume growth on M9 minimal medium supplemented with either xylose or glucose as a control after overnight growth on glucose by measuring the regrowth lag time as previously described (40). On glucose, the regrowth lag times were homogeneous, and all the strains resumed growth with an average lag of 0.80 ± 0.24 h, except for the E. coli BL21(DE3) strain, which immediately started to grow again (lag time = 0.11 ± 0.19 h) (Fig. 1A). The maximum growth rates (μ_max_) of each strain on glucose were also quantified and ranged from 0.62 to 0.95 h^−1^ (Fig. 1A). The regrowth lag times required to adapt to xylose proved to be significantly longer and varied more between strains: the lag times ranged from 1.8 ± 0.14 h lag for E. coli BW25113 to as high as 5.8 ± 0.72 h for strain E2348/69, while the BL21(DE3) strain, for an unknown reason, resumed growth even faster than its maximum growth rate after the switch (Fig. 1A). Maximum growth rates on xylose also proved to be more variable between strains than on glucose and ranged from 0.22 to 0.91 h^−1^ (Fig. 1A). No correlation was found between the maximum growth rates and regrowth lag times, either during the resumption of growth on glucose or on xylose (Fig. 1B). This indicates that growth rates and lag times are not linked. We also compared the regrowth lags resulting from resumption of growth on xylose after overnight growth on glucose with the diauxic lag observed during a diauxic growth on a glucose-xylose mix (Fig. 1C). The diauxic lags were about 6 times shorter, but the two types of lags were correlated. In conclusion, lag duration appears to be a specific trait of the strains and can last for more than 5 h.

**TABLE 1:** Escherichia coli strains used in the study

Strain	Phylogroup	Description	Reference or source
BW25113	A	K-12 derivative laboratory strain	55
MG1655	A	K-12 derivative laboratory strain	55
BL21(DE3)	A	Commercial strain for heterologous expression	New England BioLabs (catalog no. C2527)
BW30270	A	K-12 derivative laboratory strain	56
E22	B1	Rabbit enteropathogenic strain	57
S5Vir	B1	Sheep enteropathogenic strain	58
SP15	B2	Commensal strain from human feces	59
M1/5	B2	Commensal strain from human feces	60
Nissle 1917	B2	Human nonpathogenic probiotic strain	61
E2348/69	B2	Human prototype strain to study enteropathogenic E. coli biology (HG2)	62
LF82	B2	Human strain isolated from a patient with Crohn’s disease (HG2)	63
CA244	B2	K-12 derivative laboratory strain	64
1404	C	Isolated from a septicemic calf	65

### XylR overexpression reduces the duration of the regrowth lag.

**FIG 2:**
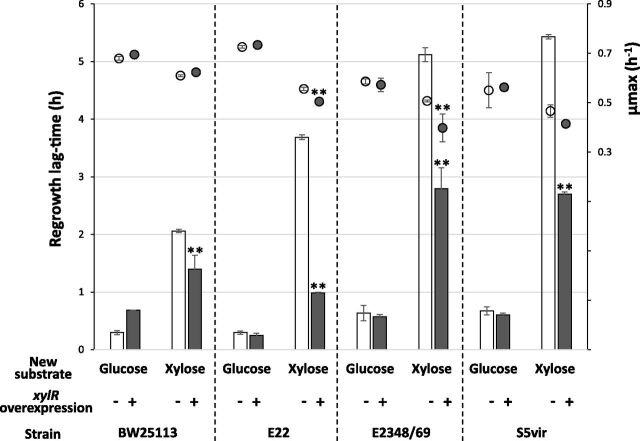
XylR overexpression reduces regrowth lag time during the glucose-xylose shift of different E. coli strains. Strains carrying the pMET219 plasmid as a control (white symbols) or the same backbone plasmid with *xylR* expression under the control of the *cysG* constitutive promoter (pMET219_xylR; gray symbols), were grown like those shown in Fig. 1A (9 biological replicates but for strain E22 without *xylR* overexpression where *n* = 6; ** indicates a significant *P* value of <0.01 compared to the control strain not overexpressing *xylR*). The bars represent the lag time (in hours) before growth resumed. The circles represent the growth rates.

XylR is a crucial transcription factor to grow on xylose. To study the relationship between XylR expression and the duration of the regrowth lag, we overexpressed it in four of the E. coli wild-type strains (BW25113, E22, E2348, and S5Vir). These strains were transformed with the plasmid pMET219_xylR that contains or does not contain the *xylR* gene under the control of the constitutive E. coli *cysG* promoter (41). Expression of *xylR* in cells harboring this plasmid will therefore be constant and not coupled with carbon catabolite repression or with the presence of xylose. As shown in Fig. 1, the ability of the strains to resume growth on glucose or on xylose was assessed after overnight growth on glucose (Fig. 2). No significant growth changes were observed with the pMET219_xylR plasmid compared to the pMET219 plasmid, but there was a slight decrease in strains E22 and E2348/69 on xylose only (Fig. 2). All the strains containing the pMET219_xylR plasmid showed a significantly reduced regrowth lag time when resuming growth on xylose compared to the strain containing the empty control plasmid pMET219. This reduction was 13% in the BW25113 strain (which naturally presents a short lag time), 44% in S5Vir, 55% in E2348/69, and up to 60% in E22. No significant reduction in the lag duration was observed in the controls when switched to glucose. Our results thus show that *xylR* overexpression is sufficient to reduce the regrowth lag time of E. coli strains resuming growth on xylose, without necessarily affecting the growth rate.

### XylR titration extends the regrowth lag.

**FIG 3:**
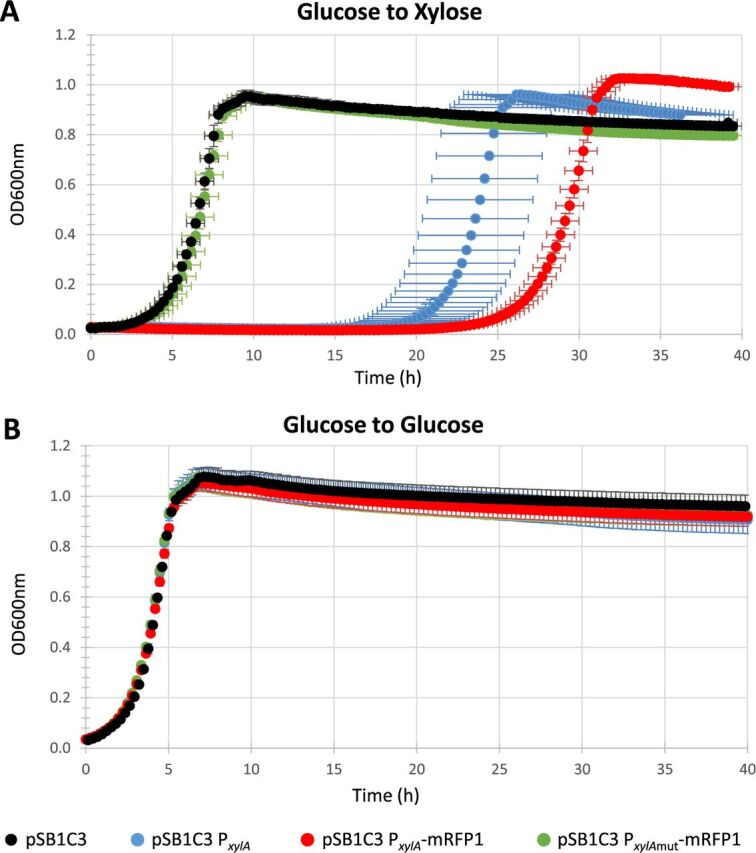
XylR titration modifies regrowth lag time. The ability of the E. coli BW25113 strain to resume growth with different plasmid constructs was assessed by switching cells after overnight culture on glucose to a 96-well microplate filled with 200 μl of fresh M9 xylose (A) or M9 glucose (B) medium. The BW25113 strain (in black) with the empty pSB1C3 plasmid as a control, the BW25113 strain (in blue) with a pSB1C3 plasmid carrying the *xylA* promoter, the BW25113 strain (in red) with a pSB1C3 plasmid expressing the red fluorescent protein mRFP1 under the control of the *xylA* promoter, and the BW25113 strain (in green) with a pSB1C3 expressing mRFP1 under the control of a *xylA* promoter deprived of its XylR binding sites. *n* = 6 for all conditions.

Since increasing XylR concentration is sufficient to reduce the regrowth lag, reducing its probability to attach to its own promoter by decreasing its availability may lengthen the lag by preventing its positive retro-control. To test this hypothesis, we increased the number of XylR targets in the cells by multiplying the *xylA* promoter in order to create a titration of the transcription factor and hence reduce its capacity to promote its own transcription through the positive-feedback regulatory loop. To this end, the *xylA* promoter was placed on the pSB1C3 high-copy-number plasmid and introduced into the BW25113 strain. The capacity of this strain to resume growth on xylose was assessed after overnight growth on glucose (Fig. 3A). As expected, there was a marked increase in the lag duration from 1.7 h for the pSB1C3 plasmid without the construct to more than 19 h for the plasmid harboring the *xylA* promoter with no impact on the maximum growth rate. The lag increased to 23 h in the *xylA* promoter driving the expression of an mRFP1 red fluorescent reporter (RFP stands for red fluorescent protein). To confirm that the increase in the regrowth lag originated specifically from the interaction between XylR and its targets on the plasmid *xylA* promoter, we mutated the two XylR binding sites in the promoter sequence. As expected, these mutations completely stopped the increase in the lag. None of these mutations affected the resumption of growth on glucose (Fig. 3B). These results show that the regrowth lag duration can be extended by titration of XylR. This result and the previous one showing that increasing XylR concentration reduces the regrowth lag duration (Fig. 2) demonstrate that the lag in resuming growth on xylose depends on XylR availability.

### Emergence of subpopulations during the glucose-to-xylose regrowth and diauxic lags.

**FIG 4:**
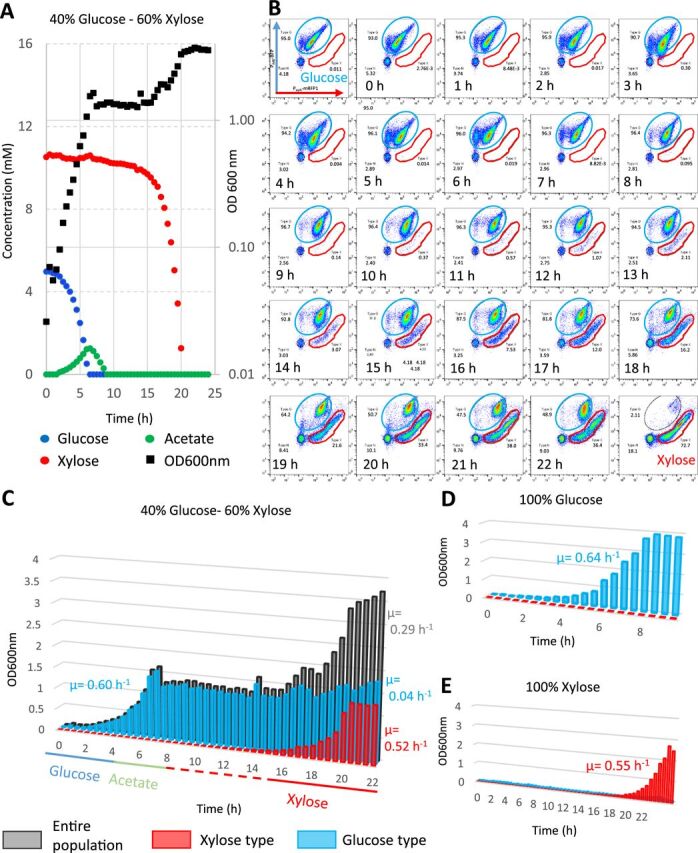
The length of the transition in the modified BW25113 strain is related to the emergence of a new subpopulation. Batch fermentations of the BW25113 strain transformed with the pSB1C3 plasmid carrying the P*_ihfB_*-BFP P*_xylA_*-mRFP1 construct were carried out in minimum medium of 90 mMeqC and sampled at 30-min intervals. (A) Kinetics of substrate consumption and production and growth of the strain in M9 medium supplemented with a 40% glucose and 60% xylose mix. (B) Cytometric profiles of the 40% glucose−60% xylose batch over time. The *y* axis displays the blue fluorescence levels in arbitrary fluorescence units (a.f.u.), and the *x* axis displays the red fluorescence levels (a.f.u.). The blue gate represents the glucose population type as seen with the 100% glucose control. The red gate represents the xylose population type as seen with the 100% xylose control. (C) The theoretical biomass of each subpopulation was extrapolated from the percentages resulting from the flow cytometry analyses during growth on the 40% glucose and 60% xylose mix (glucose type cells in blue, xylose type cells in red, and the whole population in gray). (D) Same representation with 100% glucose growth. (E) Same representation with 100% xylose growth. Others substrate ratios are presented in Fig. S2 in the supplemental material.

In the previous experiment, we managed to dramatically extend the duration of the regrowth lag to more than 19 h without affecting the maximum growth rate on xylose. We wondered why the culture took so long to resume growth. On the one hand, this behavior is artifactual in the BW25113 strain since it is due to the presence of the plasmid. On the other hand, long lags up to 6 h were observed in other genetic backgrounds (Fig. 1). Since the presence of the plasmid allows us to investigate the capacity of individual cells to express the *xylAB* operon by flow cytometry and since bimodality has been reported for the *xylAB* promoter (39), we investigated the putative existence of subpopulations during glucose-to-xylose adaptation in the modified BW25113 strain. To this end, we used the high-copy-number plasmid described above that expresses the red fluorescent protein mRFP1 under the control of the *xylA* promoter (pSB1C3 P*_xylA_*-mRFP1) so that the reporter is expressed when the cells metabolize xylose (see Fig. S1 in the supplemental material). We also added a P*_ihfB_*-mTagBFP construct to constitutively express the blue fluorescent protein (BFP) under the control of the *ihfB* promoter (41). This facilitated the detection of cells by flow cytometry. We also integrated the P*_ihfB_*-mTagBFP construct in the bacterial chromosome but the fluorescence levels were insufficient to track subpopulations (data not shown). The pSB1C3 P*_xylA_*-mRFP1 P*_ihfB_*-mTagBFP plasmid was inserted into the BW25113 strain and grown in controlled aerobic batch conditions. Cultures with different glucose-to-xylose ratios totaling up to 90 mMeqC (see “Growth media” in Materials and Methods) were performed, as well as 100% glucose or 100% xylose controls in M9 medium (see Fig. 4A for the 40% glucose and 60% xylose mix; see Fig. S2 for other mix ratios). With the 40% glucose and 60% xylose mix (Fig. 4A), a first exponential growth phase was observed associated with glucose consumption and acetate production. After the glucose was exhausted, acetate catabolism was observed, with no detectable biomass production, as described in reference 42. A transition phase lasting more than 10 h was then observed before the second exponential growth associated with xylose consumption, indicating that the plasmid construct extends the diauxic lag (Fig. 4A). This was also observed with the 100% xylose condition, indicating that the plasmid construct also increases the regrowth lag from glucose to xylose (Fig. 4E).

10.1128/mBio.02938-20.1 FIG S1 Median fluorescence intensity of E. coli BW25113 strain carrying the dual fluorescence plasmid. Download FIG S1, PDF file, 0.5 MB. Copyright © 2020 Barthe et al. 2020 Barthe et al. This content is distributed under the terms of the Creative Commons Attribution 4.0 International license.

10.1128/mBio.02938-20.2 FIG S2 Kinetics of substrate consumption and production and growth of the strain in M9 medium supplemented with a 100% glucose, 80% glucose and 20% xylose mix, 60% glucose and 40% xylose mix, 40% glucose and 60% xylose mix, 80% glucose and 20% xylose mix, or 100% xylose. Download FIG S2, PDF file, 0.5 MB. Copyright © 2020 Barthe et al. 2020 Barthe et al. This content is distributed under the terms of the Creative Commons Attribution 4.0 International license.

Flow cytometry analyses were performed with two-dimensional representation of the red inducible fluorescence versus the blue constitutive fluorescence (Fig. 4B). The cytometric profiles are displayed for growth on glucose or on xylose (the first and last graph, respectively), every hour during growth on the 40% glucose and 60% xylose mix. A constant patch of nonfluorescent cells and debris (in the black circles) representing less than 10% of the signals was always observed and hence no longer taken into consideration. The profiles obtained with growth on single substrates revealed that the strain profiles differed on glucose (in the blue gate) and on xylose (in the red gate). Although these profiles were not highly focused, probably due to cell-to-cell variations in the plasmid copy number, combined analyses of the two reporters provided a discriminating flow cytometry signature of cells growing on glucose or on xylose. These specific cell signatures were then used for population identification and quantification in the different sugar mixtures.

During growth on glucose and until its exhaustion after 7 h, the cytometric profile of the population was homogeneous and the population exhibited the same fluorescence pattern as the 100% glucose control (Fig. 4B). After glucose exhaustion (7 h), a new subpopulation gradually emerged with a cytometric pattern that matched the one obtained on the 100% xylose control. Very interestingly, the new xylose-specific population did not appear to derive from a global shift of the whole population but to emerge from individual cells following glucose exhaustion. Figure 4C shows the theoretical biomass of each subpopulation extrapolated from the percentages resulting from the flow cytometry data. This reveals the exponential growth of the new “xylose” subpopulation, with a growth rate of 0.52 h^−1^ equivalent to what was observed with the single population growing in the 100% xylose control (0.55 h^−1^) (Fig. 4E). Consistent results were obtained with other mixes (Fig. S3), showing that the glucose/xylose ratio is not impacting the phenomenon. The original population that grew on glucose grew weakly or not at all during the xylose consumption phase (0.04 h^−1^). The same representations for the 100% glucose control and the 100% xylose controls are shown in Fig. 4D and E with a homogeneous population profile on glucose and a long regrowth lag before the xylose subpopulation emerges on xylose. In conclusion, by titrating XylR availability, we observed the emergence of a subpopulation able to grow on xylose during the observed regrowth and diauxic phases, while the initial subpopulation that developed on glucose appeared to remain stable. The new emerging subpopulation thus starts from a small number of exponentially growing individuals, and not from the adaptation of the whole population. This behavior explains why the lags can last for many hours in the modified BW25113, i.e., until the new population is big enough to be detected.

10.1128/mBio.02938-20.3 FIG S3 The biomass of each subpopulation extrapolated from the percentages resulting from the flow cytometry analyses during the growth on 100% glucose, 80% glucose and 20% xylose mix, 60% glucose and 40% xylose mix, 40% glucose and 60% xylose mix, 80% glucose and 20% xylose mix, or 100% xylose. Download FIG S3, PDF file, 0.8 MB. Copyright © 2020 Barthe et al. 2020 Barthe et al. This content is distributed under the terms of the Creative Commons Attribution 4.0 International license.

### Xylose exposure creates a memory effect through XylR accumulation.

**FIG 5:**
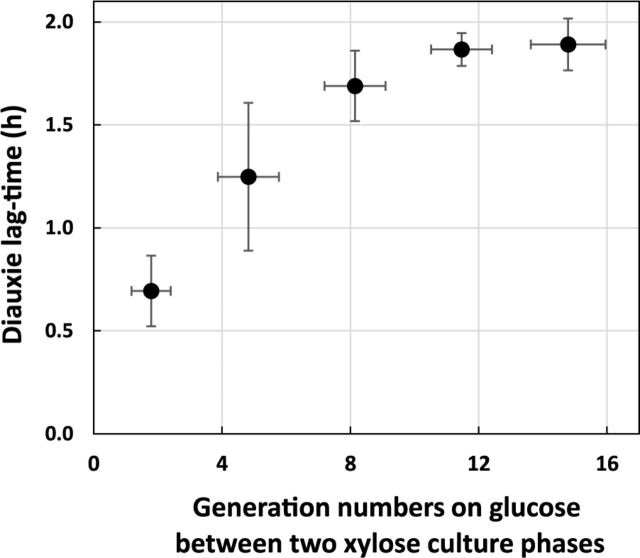
Previous exposure to xylose creates a memory effect. The strain with the titration plasmid was grown on xylose and switched to a glucose-xylose mix on microplates with different concentrations of inoculant so that the number of generations on glucose differs between two growth phases on xylose. The duration of the diauxic lags are represented as a function of the number of generations on glucose. For each condition, four replicates were performed. *n* = 4 for all generation numbers but the highest (*n* = 2).

Since the glucose-to-xylose adaptation is controlled by the XylR availability in cells, growth on xylose should produce cells with a high XylR copy number which, in turn, should allow growth to resume faster when xylose is again usable, i.e., through a memory effect. This hypothesis was tested by using the titrated strain BW25113 with the pSB1C3 P*_xylA_*-mRFP1 P*_ihfB_*-mTagBFP plasmid and its extended lags. The strain was grown overnight on xylose (or glucose as a control) before switching to fresh medium with a mix of 12.5% glucose and 87.5% xylose in microplates with inoculation concentrations ranging from an optical density at 600 nm (OD_600_) of 0.1 to 0.00001. These different concentrations of inoculant allowed the cells to perform a range of generation numbers from 1.8 to 14.8 on glucose before the final growth phase on xylose. We hypothesized that a more recent exposure to xylose would enable faster resumption of growth when xylose again became usable (i.e., once the glucose was exhausted). In this way, we measured the diauxic time to resume growth on xylose following the glucose growth phase (Fig. 5). The diauxic lag appears much shorter using microplates than in bioreactors (as in Fig. 4A). For the five cultures pregrown on xylose and switched at different inoculum rates to the glucose-xylose mix, we found a clear positive correlation between the diauxic lag durations and the number of generations on glucose since the last exposure to xylose, indicating that even a couple of generations on glucose seems efficient. No impact on diauxic time was observed in the control with pregrowth on glucose before switching to the carbon mix (data not shown). The proportionality between the diauxic lag times and the generation number on glucose is likely related to the XylR dilution in the daughter cells. This clearly identifies a “memory effect” when cells have been preexposed to xylose: XylR abundance in the cells that have been grown on xylose decreases when cells are not producing more XylR, as when grown on glucose, but remains higher than in cells that have never been exposed to xylose, hence facilitating the switch toward xylose consumption when this sugar is again available.

### Mathematical modeling of the transition highlights the effect of XylR quantity on the duration of the diauxic lag.

**FIG 6:**
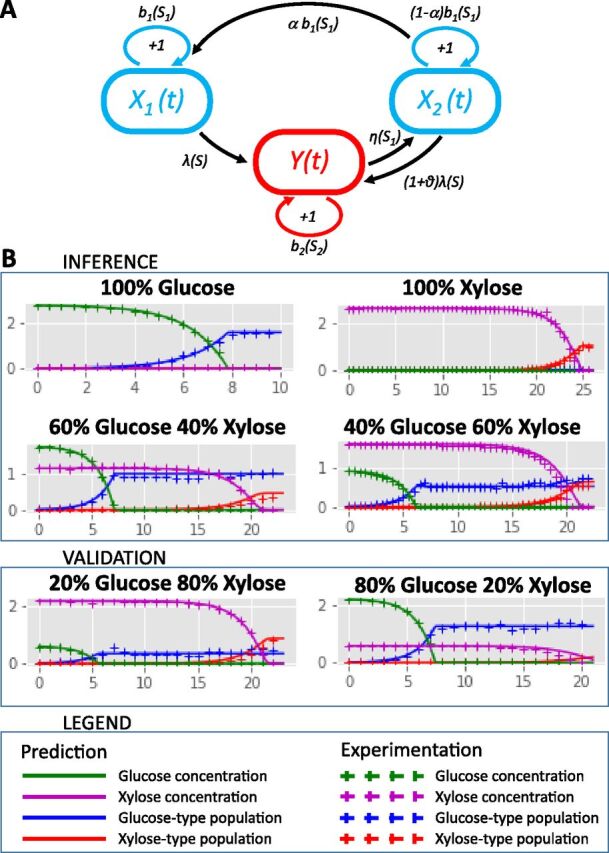
Modeling subpopulation behavior. (A) Scheme of the stochastic model. The diagram shows individual growth and transitions between cell types in the population. X_1_ corresponds to glucose consumers with the basal level of XylR, Y to xylose consumers with a high level of XylR, and X_2_ to glucose consumers that previously grew on xylose with an initially high but decreasing level of XylR. We assume that these mechanisms occur after a random time distributed according to an exponential law with the corresponding rates. Indeed, an individual that grows on glucose (class X_1_ or X_2_) divides at rate *b*_1_(*S*_1_) or switches to xylose consumption at rate λ(*S*) and (1+θ)λ(*S*), respectively. Specifically, in the X_2_ compartment, where cells have many *xylR* copies, each individual can give birth to a cell of the compartment X_1_ with probability 0 < α < 1 because of *xylR* dilution. In addition, an individual growing on xylose (class Y) divides at rate *b*_2_(*S*_2_) or switches to glucose consumption (class X_2_) at rate η(*S*_1_), if glucose is abundant. (B) Validation and predictions of the model (solid lines) compared to the experimental data (crosses) during growth on six glucose-xylose mixes (glucose cell type in blue, xylose cell type in red, glucose in green, and xylose in violet). The conditions 80% glucose−20% xylose and 20% glucose−80% xylose were not used to estimate the model parameters. The lines represent predictions, whereas in the other conditions, they represent validation.

To formalize the mechanism linking XylR concentration, subpopulation, and the diauxic lag, a modeling approach was developed based on the results of cytometry obtained with the fluorescent reporter genes. The objective was to be able to predict the behavior of the nonfluorescent wild type for which modeling of the system was mandatory. The major phenomenon to be modeled is the appearance of the “xylose” subpopulation cells. From our experimental observations, they are clearly present 1.5 h after exhaustion of the glucose (8 h in Fig. 4B), and are very likely present in very small numbers soon after glucose exhaustion when the carbon catabolite repression is switched off. Based on our previous finding concerning the major role played by XylR concentration in the lag duration, our main hypothesis is that low XylR availability results in a low probability to trigger the XylR positive-feedback loop and hence, to switch to xylose consumption. We decided to opt for a stochastic model to account for the probability that a very limited number of cells is able to resume growth on xylose at the end of glucose consumption. This scenario is backed up by proteomic data showing that XylR is present at a rate of 1 ± 0.4 copy per cell during growth on glucose and at 57 ± 8 copies per cell on xylose (supplementary data file in reference 35). Thus, after glucose is exhausted, the single copy of XylR needs to find its target on the *xylAB* promoter to enable growth on xylose. Knowing that the pUC19-derived pSB1C3 is a high-copy-number plasmid, it seemed obvious that target multiplication reduces the probability of triggering the loop. Starting from these hypotheses, we built the model presented in Fig. 6A and fully described in Text S1 in the supplemental material. Basically, the model allows a population X_1_ with a low level of XylR to grow on glucose and to switch to growth on xylose only if glucose is exhausted and xylose is present. In this situation, cells from population X_1_ will turn at rate λ into cells with Y population features (i.e., xylose consumers with a high level of available XylR). Return to the initial X_1_ state is possible in the model if glucose again becomes available: the cells will transit through an X_2_ state characterized by a decrease in XylR content by dilution through cell division until the cells reach the XylR level of the X_1_ population. For a population close to a billion cells, the stochastic model is approximated by a deterministic dynamic system, making it easier to calibrate the parameters (Text S1). In other words, the core of the model remains stochastic to capture the appearance of the new subpopulation, but it could be approximated to a deterministic model once the number of individuals is high enough. This model was calibrated with data from culture with 100% glucose, 100% xylose, a 60% glucose-40% xylose mix and a 40% glucose-60% xylose mix. It accurately predicted these four conditions in terms of subpopulation distribution and substrate consumption as well as the data not used for the calibration from cultures with 80% glucose-20% xylose and 20% glucose-80% xylose mixes (Fig. 6B). Parameter calibrations made it possible to quantify the rate of appearance of xylose consumers (Y) in the original population (X_1_) after glucose exhaustion to 2.02 × 10^−04^ h^−1^, i.e., about 2 cells over 10,000 per hour.

10.1128/mBio.02938-20.8 TEXT S1 Mathematical modeling of an E. coli batch culture on glucose and xylose. Download Text S1, PDF file, 0.1 MB. Copyright © 2020 Barthe et al. 2020 Barthe et al. This content is distributed under the terms of the Creative Commons Attribution 4.0 International license.

Next, the model was used to simulate the behavior of the wild-type strain throughout the diauxie; the structure of the model was retained, but the values of growth and substrate consumption rates specific to the strain were recalibrated. Very interestingly, the subpopulation balance appeared to be the inverse of what we observed with the strain carrying the pSB1C3 P*_xylA_*-mRFP1 P*_ihfB_*-mTagBFP plasmid (Fig. S4). Indeed, without the plasmid, the individual rate of appearance of xylose consumers from the original population after glucose exhaustion was 2.09 h^−1^, i.e., about 8,760 cells to over 10,000 per hour. This result is of particular interest, since it makes it possible to predict the behavior of the wild-type strain without the bias created by markers. The model allowed us to estimate that, by the time xylose was exhausted, 99.46% of the X_1_ population have shifted to xylose consumption. In a population of close to a billion cells, this predicts that about 5.4 million cells will remain in a dormant-like state.

10.1128/mBio.02938-20.4 FIG S4 Experimental and prediction data for the wild-type strain BW25113 on a glucose-xylose mix. Download FIG S4, PDF file, 0.6 MB. Copyright © 2020 Barthe et al. 2020 Barthe et al. This content is distributed under the terms of the Creative Commons Attribution 4.0 International license.

## DISCUSSION

Lag times have long been considered adaptation periods for a microbial population to use a new resource. The recent interest of the microbiologist community in single cell analysis revealed a more complex reality concerning these lag phases. Here, we demonstrated that the main parameter governing the length of the regrowth or diauxic lag phases is not a temporal requirement to produce new machinery for growth on the following substrate but the availability of a specific transcription factor. In the glucose-xylose model we studied here, XylR accumulation is the master molecular switch governing the metabolic fate of the cells. Consequently, titration of XylR extends lag time, while overexpression of XylR reduces it. These effects are limited to lag durations and do not affect the maximum growth rates in general, very likely because XylR is not needed on glucose while its regulatory loop ensures that it is not limiting on xylose.

This positive-feedback loop of XylR on its own expression is the centerpiece of the system. It enables the creation of the bimodality in the cell population reported by Afroz et al. (39), with some of the cells able to trigger the loop and thus shift to full growth on xylose while the others fail to do so and remain in a dormant-like state. Such a regulatory design has already been described in microorganisms faced with other substrates (39, 43–46). The question that remains is what determines the metabolic fate of individual cells. Our results suggest that XylR availability is sufficient to explain all the phenotypes we observed. Yet, it would be remarkable if accumulation of a single protein could explain so much, especially in the light of all the different molecular explanations behind phenotypic heterogeneity in the substrate usage reported in the literature (12, 13, 16, 18–24). It would be interesting to study the noise in the expression of XylR and properties of the resulting distribution even if experimental XylR quantification at the single cell level remains a great experimental challenge. This could reveal the presence of cells with high levels of XylR even before glucose exhaustion but likely not large enough to trigger the loop since we never observed cells with a xylose signature during growth on glucose. Another source of heterogeneity could be the unequal distribution of transcription factor between the mother and daughter cells since the concentration of XylR is close to one copy per cell on glucose (35). Indeed, this would create cells depleted of XylR that would be unable to trigger the loop in the wild-type strain (47). Our results do not prove that parameters reported by others such as cell size, cell cycle phase at the moment of glucose exhaustion, cell age, ribosome availability, or ppGpp concentration have no effect on the adaptation from glucose to xylose. The fact that XylR overexpression does not produce regrowth lags with the same optimal duration in different strains, or the different lag durations observed between microplate and bioreactor cultures supports the hypothesis that other parameters are at stake here. It is for example rational to hypothesize a role for carbon catabolite repression in the process. The presence of CRP-cAMP on the promoter could be required for XylR to fix on its recognition sites or to create a slight increase in *xylR* promoter expression ensuring that XylR is over a limiting threshold to trigger the loop that most cells with the titration plasmid cannot overcome.

The model parameter estimation showed that most of the cells are not able to switch to xylose consumption in the mutant strain because of XylR titration, while the inverse situation was observed in the wild-type strain with only a small fraction of cells unable to switch. The cells that are unable to use the available substrate could be dormant cells. Such behavior has already been reported in other conditions when a population was faced with a new substrate (3, 12, 48). This was interpreted as an adaptive feature to minimize risk since the dormant cells are more resistant to environmental insults, including stresses or antibiotics (48–50). These cells are metabolically active with very slow buildup of biomass and reduced metabolite pools (50), which could match the very small growth rate observed for this subpopulation in our study. Even if the fraction of such cells is low in the wild-type strain, the model predicted that it nevertheless results in millions of individuals in the bacterial population as a whole. This reinforces the hypothesis that metabolic switches are related to antibiotic resistance (48).

To our knowledge, this is the first work exploring phenotypic heterogeneity in the use of substrate during diauxic batch fermentation of a monoclonal population in infinitely stirred bioreactor. Most studies have used microfluidic devices and/or manual switching of the cells from one condition to another, both being not ideal for analyzing responses to environmental changes that happen as a consequence of growth (51). This allows confirming that phenotypic heterogeneity in the use of the substrate observed at the scale of a few individual cells is also true in large populations growing naturally. Likewise, phenotypic heterogeneity has been reported to have an incidence on metabolic adaptation of a population from glycolytic to gluconeogenic substrates (13). Here, we report that the same incidence could be true for metabolic adaptation from glycolytic to glycolytic substrates.

Several applications of this work can be envisaged. It now appears possible to optimize lags to ensure the profitability or sustainability of biotechnological processes, for example, by reducing the length of lag phases. Our results point to two levers that could achieve this. The first is by preexposing the cells to the substrates they will use during the process to take advantage of the memory effect. The effect has indeed been observed with a variety of substrates (16, 22), and this solution appears to be both cheap and effective. The second solution is modifying the positive-feedback loop through overexpression of the limiting transcription factor as we did here or modifying the regulatory network. Indeed, such loops are not limited to the use of xylose but are reported with increasing frequency in adaptive phenomena (16, 17, 52, 53). Other levers very likely exist. For example, we observed here that the transition is faster during microplate culture than during well-controlled bioreactor culture even though we have no explanation for this as yet. Conversely, it would be interesting to extend the diauxic lag phase since this could promote the formation of dormant cells, which could be a key factor in ensuring the robustness of the strain during challenging bioprocesses or for strain storage. Finally, the extended duration of the glucose-xylose transition could be of fundamental interest to study the properties of dormant cells.

Last, it is now clear that lag phases (as well as the so-called “stationary phases”) are far more complex than originally thought by Jacques Monod in 1942 (54). Until the last decade, most interest focused on the exponential phases, and we are only now beginning to become aware of the subtle behavior of cells that are not growing, from the organization of subpopulations to persistence and maintenance costs.

## MATERIALS AND METHODS

All data are accessible on the INRAE platform (https://doi.org/10.15454/XWCMZE).

### Strains and plasmids.

All the strains used in this study are listed in Table 1.

All the plasmids used in this study are listed in Table 2. All the plasmids based on the pSB1C3 backbone were constructed through digestion and ligation. The *xylA* promoter was obtained by amplification of 800 bp of the regulatory area of the gene upstream of its ATG from genomic DNA of the BW25113 strain. The *xylA* mutated promoter was designed as shown in Fig. S5 in the supplemental material and obtained as a synthetic gene (Eurofins). EcoRI and XbaI restriction sites at the 5′ end and SpeI restriction site at the 3′ end were used for cloning on the chassis plasmid. Primers (Fig. S6) were designed from the sequences in the Ecocyc database obtained from Eurofins (France). For the construction of the double fluorescence plasmid, the pSB1C3 P*_ihfBA_*-mTagBFP was digested with EcoRI and SpeI to be ligated in the pSB1C3 P*_xylA_*-mRFP1 linearized with EcoRI and XbaI digestion. The plasmids based on pBR322 were constructed by DNA HiFi assembly (New England Biolabs). Fragments were amplified from E. coli DNA or plasmid purification with 20 bp of homology between each fragment. For all constructs, transformants were selected on LB plates containing 40 mg/liter of chloramphenicol. The plasmids were checked by sequencing (Eurofins). Plasmids were then transformed into E. coli BW25113 strain.

**TABLE 2:** Plasmids used in this study

Plasmid	Relevant characteristics	Reference
pSB1C3 mRFP1	Cloning vector of this study. pUC19-derived pMB1 ori, chloramphenicol resistance, with mRFP1 (FPbase identifier [ID] 5YCFA) coding sequence	iGEM part BBa_E1010
pSB1C3 mTagBFP	Cloning vector. pUC19-derived pMB1 ori, chloramphenicol resistance, with mTagBFP (FPbase ID BFJKS) coding sequence	iGEM part BBa_K592100
pSB1C3 P*_ihfBA_*-mTagBFP	pSB1C3 plasmid containing mTagBFP under the control of *ihfB* promoter	This study
pSB1C3 P*_xylA_*-mRFP1	pSB1C3 plasmid containing mRFP1 under the control of *xylA* promoter	This study
pSB1C3 P*_xylA_-mut*-mRFP1	pSB1C3 plasmid containing mRFP1 under the control of a mutated *xylA* promoter, mutations were introduced in *xylR* binding sites	This study
pSB1C3 P*_xylA_*-mRFP1 P*_ihfB_*-mTagBFP	pSB1C3 plasmid containing mTagBFP under the control of *ihfB* promoter and mRFP1 under the control of *xylA* promoter	This study
pSB1C3 P*_xylA_*	pSB1C3 plasmid containing *xylA* promoter	This study
pMET219	Plasmid pBR322 with tetR but ampR replaced by cmR. Negative control of the plasmid pMET219	Gift from Sébastien Nouaille
pMET219 P*_cysG_*-*xylR*	Plasmid pMET219 with tetR replaced by *xylR* under control of *cysG* promoter	This study

10.1128/mBio.02938-20.5 FIG S5 Sequence of the *xylA* mutated promoter used in this study. Download FIG S5, PDF file, 0.5 MB. Copyright © 2020 Barthe et al. 2020 Barthe et al. This content is distributed under the terms of the Creative Commons Attribution 4.0 International license.

10.1128/mBio.02938-20.6 FIG S6 List of primers used to build the plasmids used in this study. Download FIG S6, PDF file, 0.5 MB. Copyright © 2020 Barthe et al. 2020 Barthe et al. This content is distributed under the terms of the Creative Commons Attribution 4.0 International license.

### Growth media.

The cultures were grown in M9 medium containing 33.7 mM Na_2_HPO_4_, 22 mM KH_2_PO_4_, 8.55 mM NaCl, 9.35 mM NH_4_Cl, 1 mM MgSO_4_, 0.3 mM CaCl_2_, 13.4 mM EDTA, 3.1 mM FeCl_3_·6H_2_O, 0.62 mM ZnCl_2_, 76 μM CuCl_2_·2H_2_O, 42 μM CoCl_2_·2H_2_O, 162 μM H_3_BO_3_, 8.1 μM MnCl_2_·4H_2_O, 1 μg · liter^−1^ thiamine and carbon sources at 90 mMeqC (100% glucose, 100% xylose, 80% glucose plus 20% xylose, 60% glucose plus 40% xylose, 40% glucose plus 60% xylose, or 20% glucose plus 80% xylose). For the culture of strains containing plasmids, M9 medium was supplemented with 20 mg · liter^−1^ chloramphenicol.

### Microplate cultivation.

Cells were pregrown overnight in 5-ml tubes with M9 medium with glucose (M9 glucose medium) at 37°C with agitation at 150 rpm. Overnight cultures were centrifuged at 5,000 rpm for 3 min, and pellets were suspended in M9 glucose medium or M9 xylose medium at an initial OD_600_ of 0.05. The 96-well microplates were inoculated in triplicate with 200 μl of the cell suspensions or 200 μl of medium as a control. Microplates with lids were read at OD_600_ at 17-min intervals for 24 h with a Versamax microplate reader (Molecular Devices, USA). Maximum growth rates μ_max_ were determined during the exponential growth phase and estimated using the coefficient of the linear regression of the curve representing ln OD versus time.

Regrowth lag times were considered the difference in time needed to reach the same OD point in the exponential phase between growth with a time lag and growth that started immediately at the maximum growth rate, as defined by Enjalbert et al. (40). Diauxic lag was considered the time lost in comparison to hypothetical growth in which the first exponential phase is immediately followed by a second exponential phase. Formulas and demonstrations of how the lag was calculated are provided in Fig. S7.

10.1128/mBio.02938-20.7 FIG S7 Demonstration of the diauxic lag formula. Download FIG S7, PDF file, 0.5 MB. Copyright © 2020 Barthe et al. 2020 Barthe et al. This content is distributed under the terms of the Creative Commons Attribution 4.0 International license.

Statistical validation was based on paired two-tailed Student’s *t* tests, assuming equal or unequal variances according to the F test. Means were calculated from three biological replicates with three technical replicates each (*n* = 9).

### Batch cultivation.

Bioreactor batch cultures were performed in a Sartorius Biostat B plus bioreactor in 1 liter of M9 medium with 90 mMeqC of glucose and/or xylose mixes. The temperature was set at 37°C and pH at 7. Nonlimiting aeration conditions were obtained with an airflow set at 0.35 liter · min^−1^ and adaptation of stirring to maintain pO_2_ of >20%. Growth was assessed by OD_600_ measurement at 30-min intervals with a LibraS4 spectrophotometer (Biochrom, UK). Glucose, xylose, and acetate concentrations were quantified by high-performance liquid chromatography (HPLC) (Agilent, USA) with an HPX 87H ion exclusion column (Bio-Rad) as described in reference 66.

### Flow cytometry.

Cells were sampled at 30-min intervals and immediately frozen at –20°C; tests showed that freezing has no influence on the level of fluorescence. After thawing and diluting in filtered 0.9% NaCl to obtain a cell concentration of 1 × 10^6^ cells · ml^−1^, fluorescence intensities were measured with a Masquant VYB cytometer (Miltenyi Biotec, Germany) equipped with a 561-nm yellow laser for excitation of mRFP1 and a 405-nm purple laser for mTagBFP. Data acquisition was set at 40,000 events per sample. FlowJo X software was used for cytometry data analysis. A gate was created in the dot plot of the forward scatter channel (FSC-H) versus the side scatter channel (SSC-H) to distinguish bacteria from technical noise. A second gate was created in the dot plot of the SSC-H versus the SSC-A to select single cells. The mTagBFP fluorescence emissions were analyzed with the V1-H channel (BP450/25 nm) and the mRFP1 emissions with the Y2-H channel (BP615/10 nm). The OD_600_ subpopulation was extrapolated from the total OD_600_ population and the percentage of each subpopulation obtained in flow cytometry, based on blue and red fluorescence.

### Mathematical modeling.

A detailed description of the model, including its rationale, parameters, and code, is provided in Text S1 in the supplemental material.

### Memory effect experiment.

Cells were pregrown overnight in tubes containing 5 ml of M9 glucose medium or M9 xylose medium at 37°C with agitation (150 rpm). Cells were centrifuged at 5,000 rpm for 3 min, and pellets were suspended and inoculated when they reached an OD_600_ of 0.1 in 100-ml Erlenmeyer flasks containing 20 ml of M9 glucose medium or M9 xylose medium. Once in the exponential phase (OD_600_ of ca. 1), the cultures were diluted (theoretical OD_600_s of 0.1, 0.01, 0.001, 0.0001 and 0.00001) and inoculated in duplicate in 96-well microplates in 200 μl of M9 glucose, M9 xylose, or M9 medium with 12.5% glucose and 87.5% xylose. Wells containing 200 μl of medium with no cells were used as a control. Microplates with a lid were read at OD_600_ at 17-min intervals for 24 h with a Versamax microplate reader (Molecular Devices, USA). Two biological replicates each with two technical replicates were performed.

## References

[1] Monod J. 1949. The growth of bacterial cultures. Annu Rev Microbiol 3:371–394. doi:10.1146/annurev.mi.03.100149.002103.

[2] Madar D, Dekel E, Bren A, Zimmer A, Porat Z, Alon U. 2013. Promoter activity dynamics in the lag phase of Escherichia coli. BMC Syst Biol 7:136. doi:10.1186/1752-0509-7-136. 24378036 PMC3918108

[3] Jõers A, Tenson T. 2016. Growth resumption from stationary phase reveals memory in Escherichia coli cultures. Sci Rep 6:24055. doi:10.1038/srep24055. 27048851 PMC4822139

[4] Chu D, Barnes DJ. 2016. The lag-phase during diauxic growth is a trade-off between fast adaptation and high growth rate. Sci Rep 6:25191. doi:10.1038/srep25191. 27125900 PMC4850433

[5] Aidelberg G, Towbin BD, Rothschild D, Dekel E, Bren A, Alon U. 2014. Hierarchy of non-glucose sugars in Escherichia coli. BMC Syst Biol 8:133. doi:10.1186/s12918-014-0133-z. 25539838 PMC4304618

[6] Ammar EM, Wang X, Rao CV. 2018. Regulation of metabolism in Escherichia coli during growth on mixtures of the non-glucose sugars: arabinose, lactose, and xylose. Sci Rep 8:609. doi:10.1038/s41598-017-18704-0. 29330542 PMC5766520

[7] Görke B, Stülke J. 2008. Carbon catabolite repression in bacteria: many ways to make the most out of nutrients. Nat Rev Microbiol 6:613–624. doi:10.1038/nrmicro1932. 18628769

[8] Wolfe AJ. 2005. The acetate switch. Microbiol Mol Biol Rev 69:12–50. doi:10.1128/MMBR.69.1.12-50.2005. 15755952 PMC1082793

[9] Traxler MF, Chang D-E, Conway T. 2006. Guanosine 3′,5′-bispyrophosphate coordinates global gene expression during glucose-lactose diauxie in Escherichia coli. Proc Natl Acad Sci U S A 103:2374–2379. doi:10.1073/pnas.0510995103. 16467149 PMC1413745

[10] Boulineau S, Tostevin F, Kiviet DJ, ten Wolde PR, Nghe P, Tans SJ. 2013. Single-cell dynamics reveals sustained growth during diauxic shifts. PLoS One 8:e61686. doi:10.1371/journal.pone.0061686. 23637881 PMC3640066

[11] van Heerden JH, Wortel MT, Bruggeman FJ, Heijnen JJ, Bollen YJM, Planqué R, Hulshof J, O’Toole TG, Wahl SA, Teusink B. 2014. Lost in transition: start-up of glycolysis yields subpopulations of nongrowing cells. Science 343:1245114. doi:10.1126/science.1245114. 24436182

[12] Solopova A, van Gestel J, Weissing FJ, Bachmann H, Teusink B, Kok J, Kuipers OP. 2014. Bet-hedging during bacterial diauxic shift. Proc Natl Acad Sci U S A 111:7427–7432. doi:10.1073/pnas.1320063111. 24799698 PMC4034238

[13] Kotte O, Volkmer B, Radzikowski JL, Heinemann M. 2014. Phenotypic bistability in Escherichia coli’s central carbon metabolism. Mol Syst Biol 10:736. doi:10.15252/msb.20135022. 24987115 PMC4299493

[14] Nikolic N, Schreiber F, Dal Co A, Kiviet DJ, Bergmiller T, Littmann S, Kuypers MMM, Ackermann M. 2017. Cell-to-cell variation and specialization in sugar metabolism in clonal bacterial populations. PLoS Genet 13:e1007122. doi:10.1371/journal.pgen.1007122. 29253903 PMC5773225

[15] Bertrand RL. 2019. Lag phase is a dynamic, organized, adaptive, and evolvable period that prepares bacteria for cell division. J Bacteriol 201:e00697-18. doi:10.1128/JB.00697-18. 30642990 PMC6416914

[16] Kaiser M, Jug F, Julou T, Deshpande S, Pfohl T, Silander OK, Myers G, van Nimwegen E. 2018. Monitoring single-cell gene regulation under dynamically controllable conditions with integrated microfluidics and software. Nat Commun 9:212. doi:10.1038/s41467-017-02505-0. 29335514 PMC5768764

[17] Takhaveev V, Heinemann M. 2018. Metabolic heterogeneity in clonal microbial populations. Curr Opin Microbiol 45:30–38. doi:10.1016/j.mib.2018.02.004. 29477028

[18] Martins BM, Locke JC. 2015. Microbial individuality: how single-cell heterogeneity enables population level strategies. Curr Opin Microbiol 24:104–112. doi:10.1016/j.mib.2015.01.003. 25662921

[19] Axelrod K, Sanchez A, Gore J. 2015. Phenotypic states become increasingly sensitive to perturbations near a bifurcation in a synthetic gene network. eLife 4:e07935. doi:10.7554/eLife.07935. PMC4547091 26302311

[20] Amato SM, Brynildsen MP. 2015. Persister heterogeneity arising from a single metabolic stress. Curr Biol 25:2090–2098. doi:10.1016/j.cub.2015.06.034. 26255847

[21] Yaginuma H, Kawai S, Tabata KV, Tomiyama K, Kakizuka A, Komatsuzaki T, Noji H, Imamura H. 2014. Diversity in ATP concentrations in a single bacterial cell population revealed by quantitative single-cell imaging. Sci Rep 4:6522. doi:10.1038/srep06522. 25283467 PMC4185378

[22] Lambert G, Kussell E. 2014. Memory and fitness optimization of bacteria under fluctuating environments. PLoS Genet 10:e1004556. doi:10.1371/journal.pgen.1004556. 25255314 PMC4177670

[23] Ito Y, Toyota H, Kaneko K, Yomo T. 2009. How selection affects phenotypic fluctuation. Mol Syst Biol 5:264. doi:10.1038/msb.2009.23. 19401676 PMC2683726

[24] Dal Co A, Brannon C, Ackermann M. 2018. Division of labor in bacteria. eLife 7:e38578. doi:10.7554/eLife.38578. 29957180 PMC6025956

[25] Eiteman MA, Lee SA, Altman E. 2008. A co-fermentation strategy to consume sugar mixtures effectively. J Biol Eng 2:3. doi:10.1186/1754-1611-2-3. 18304345 PMC2266900

[26] Kim SM, Choi BY, Ryu YS, Jung SH, Park JM, Kim G-H, Lee SK. 2015. Simultaneous utilization of glucose and xylose via novel mechanisms in engineered Escherichia coli. Metab Eng 30:141–148. doi:10.1016/j.ymben.2015.05.002. 26045332

[27] Sievert C, Nieves LM, Panyon LA, Loeffler T, Morris C, Cartwright RA, Wang X. 2017. Experimental evolution reveals an effective avenue to release catabolite repression via mutations in XylR. Proc Natl Acad Sci U S A 114:7349–7354. doi:10.1073/pnas.1700345114. 28655843 PMC5514714

[28] Rossoni L, Carr R, Baxter S, Cortis R, Thorpe T, Eastham G, Stephens G. 2018. Engineering Escherichia coli to grow constitutively on D-xylose using the carbon-efficient Weimberg pathway. Microbiology 164:287–298. doi:10.1099/mic.0.000611. 29458683 PMC5882109

[29] Sumiya M, Davis EO, Packman LC, McDonald TP, Henderson PJ. 1995. Molecular genetics of a receptor protein for D-xylose, encoded by the gene xylF, in Escherichia coli. Recept Channels 3:117–128. 8581399

[30] Sooriyaarachchi S, Ubhayasekera W, Park C, Mowbray SL. 2010. Conformational changes and ligand recognition of Escherichia coli D-xylose binding protein revealed. J Mol Biol 402:657–668. doi:10.1016/j.jmb.2010.07.038. 20678502

[31] Wovcha MG, Steuerwald DL, Brooks KE. 1983. Amplification of D-xylose and D-glucose isomerase activities in Escherichia coli by gene cloning. Appl Environ Microbiol 45:1402–1404. doi:10.1128/AEM.45.4.1402-1404.1983. 6344793 PMC242470

[32] Briggs KA, Lancashire WE, Hartley BS. 1984. Molecular cloning, DNA structure and expression of the Escherichia coli D-xylose isomerase. EMBO J 3:611–616. doi:10.1002/j.1460-2075.1984.tb01856.x. 6325179 PMC557396

[33] Lawlis VB, Dennis MS, Chen EY, Smith DH, Henner DJ. 1984. Cloning and sequencing of the xylose isomerase and xylulose kinase genes of Escherichia coli. Appl Environ Microbiol 47:15–21. doi:10.1128/AEM.47.1.15-21.1984. 6320721 PMC239604

[34] Song S, Park C. 1997. Organization and regulation of the D-xylose operons in Escherichia coli K-12: XylR acts as a transcriptional activator. J Bacteriol 179:7025–7032. doi:10.1128/jb.179.22.7025-7032.1997. 9371449 PMC179643

[35] Schmidt A, Kochanowski K, Vedelaar S, Ahrné E, Volkmer B, Callipo L, Knoops K, Bauer M, Aebersold R, Heinemann M. 2016. The quantitative and condition-dependent *Escherichia coli* proteome. Nat Biotechnol 34:104–110. doi:10.1038/nbt.3418. 26641532 PMC4888949

[36] Deutscher J. 2008. The mechanisms of carbon catabolite repression in bacteria. Curr Opin Microbiol 11:87–93. doi:10.1016/j.mib.2008.02.007. 18359269

[37] Shimizu K. 2013. Metabolic regulation of a bacterial cell system with emphasis on Escherichia coli metabolism. ISRN Biochem 2013:645983. doi:10.1155/2013/645983. 25937963 PMC4393010

[38] Laikova ON, Mironov AA, Gelfand MS. 2001. Computational analysis of the transcriptional regulation of pentose utilization systems in the gamma subdivision of Proteobacteria. FEMS Microbiol Lett 205:315–322. doi:10.1111/j.1574-6968.2001.tb10966.x. 11750821

[39] Afroz T, Biliouris K, Kaznessis Y, Beisel CL. 2014. Bacterial sugar utilization gives rise to distinct single-cell behaviours. Mol Microbiol 93:1093–1103. doi:10.1111/mmi.12695. 24976172 PMC4160389

[40] Enjalbert B, Cocaign-Bousquet M, Portais J-C, Letisse F. 2015. Acetate exposure determines the diauxic behavior of Escherichia coli during the glucose-acetate transition. J Bacteriol 197:3173–3181. doi:10.1128/JB.00128-15. 26216845 PMC4560281

[41] Zhou K, Zhou L, Lim Q, Zou R, Stephanopoulos G, Too H-P. 2011. Novel reference genes for quantifying transcriptional responses of Escherichia coli to protein overexpression by quantitative PCR. BMC Mol Biol 12:18. doi:10.1186/1471-2199-12-18. 21513543 PMC3110127

[42] Enjalbert B, Letisse F, Portais J-C. 2013. Physiological and molecular timing of the glucose to acetate transition in Escherichia coli. Metabolites 3:820–837. doi:10.3390/metabo3030820. 24958151 PMC3901295

[43] Ozbudak EM, Thattai M, Lim HN, Shraiman BI, van Oudenaarden A. 2004. Multistability in the lactose utilization network of Escherichia coli. Nature 427:737–740. doi:10.1038/nature02298. 14973486

[44] Bhogale PM, Sorg RA, Veening J-W, Berg J. 2014. What makes the lac-pathway switch: identifying the fluctuations that trigger phenotype switching in gene regulatory systems. Nucleic Acids Res 42:11321–11328. doi:10.1093/nar/gku839. 25245949 PMC4191413

[45] Guantes R, Benedetti I, Silva-Rocha R, de Lorenzo V. 2016. Transcription factor levels enable metabolic diversification of single cells of environmental bacteria. ISME J 10:1122–1133. doi:10.1038/ismej.2015.193. 26636554 PMC5029220

[46] Koirala S, Wang X, Rao CV. 2016. Reciprocal regulation of l-arabinose and d-xylose metabolism in Escherichia coli. J Bacteriol 198:386–393. doi:10.1128/JB.00709-15. 26527647 PMC4719453

[47] Robert L, Paul G, Chen Y, Taddei F, Baigl D, Lindner AB. 2010. Pre-dispositions and epigenetic inheritance in the Escherichia coli lactose operon bistable switch. Mol Syst Biol 6:357. doi:10.1038/msb.2010.12. 20393577 PMC2872608

[48] Amato SM, Orman MA, Brynildsen MP. 2013. Metabolic control of persister formation in Escherichia coli. Mol Cell 50:475–487. doi:10.1016/j.molcel.2013.04.002. 23665232

[49] Veening J-W, Smits WK, Kuipers OP. 2008. Bistability, epigenetics, and bet-hedging in bacteria. Annu Rev Microbiol 62:193–210. doi:10.1146/annurev.micro.62.081307.163002. 18537474

[50] Radzikowski JL, Vedelaar S, Siegel D, Ortega ÁD, Schmidt A, Heinemann M. 2016. Bacterial persistence is an active σS stress response to metabolic flux limitation. Mol Syst Biol 12:882. doi:10.15252/msb.20166998. 27655400 PMC5043093

[51] Rosenthal AZ, Qi Y, Hormoz S, Park J, Li SH-J, Elowitz MB. 2018. Metabolic interactions between dynamic bacterial subpopulations. eLife 7:e33099. doi:10.7554/eLife.33099. 29809139 PMC6025961

[52] Choi PJ, Cai L, Frieda K, Xie XS. 2008. A stochastic single-molecule event triggers phenotype switching of a bacterial cell. Science 322:442–446. doi:10.1126/science.1161427. 18927393 PMC2819113

[53] Choudhury D, Gayen K, Saini S. 2018. Dynamic control of arabinose and xylose utilization in E. coli. Can J Chem Eng 96:1881–1887. doi:10.1002/cjce.23197.

[54] Monod J. 1942. Recherches sur la croissance des cultures bactériennes. Hermann & Cie, Paris, France.

[55] Baba T, Ara T, Hasegawa M, Takai Y, Okumura Y, Baba M, Datsenko KA, Tomita M, Wanner BL, Mori H. 2006. Construction of Escherichia coli K-12 in-frame, single-gene knockout mutants: the Keio collection. Mol Syst Biol 2:2006.0008. doi:10.1038/msb4100050. PMC1681482 16738554

[56] Datsenko KA, Wanner BL. 2000. One-step inactivation of chromosomal genes in Escherichia coli K-12 using PCR products. Proc Natl Acad Sci U S A 97:6640–6645. doi:10.1073/pnas.120163297. 10829079 PMC18686

[57] Marchès O, Nougayrède J-P, Boullier S, Mainil J, Charlier G, Raymond I, Pohl P, Boury M, De Rycke J, Milon A, Oswald E. 2000. Role of Tir and intimin in the virulence of rabbit enteropathogenic Escherichia coli serotype O103:H2. Infect Immun 68:2171–2182. doi:10.1128/iai.68.4.2171-2182.2000. 10722617 PMC97401

[58] Smith A, Kaczmar A, Bamford RA, Smith C, Frustaci S, Kovacs-Simon A, O’Neill P, Moore K, Paszkiewicz K, Titball RW, Pagliara S. 2018. The culture environment influences both gene regulation and phenotypic heterogeneity in Escherichia coli. Front Microbiol 9:1739. doi:10.3389/fmicb.2018.01739. 30158905 PMC6104134

[59] Johnson JR, Johnston B, Kuskowski MA, Nougayrede J-P, Oswald E. 2008. Molecular epidemiology and phylogenetic distribution of the Escherichia coli pks genomic island. J Clin Microbiol 46:3906–3911. doi:10.1128/JCM.00949-08. 18945841 PMC2593299

[60] Martin P, Marcq I, Magistro G, Penary M, Garcie C, Payros D, Boury M, Olier M, Nougayrède J-P, Audebert M, Chalut C, Schubert S, Oswald E. 2013. Interplay between siderophores and colibactin genotoxin biosynthetic pathways in Escherichia coli. PLoS Pathog 9:e1003437. doi:10.1371/journal.ppat.1003437. 23853582 PMC3708854

[61] Revelles O, Millard P, Nougayrède J-P, Dobrindt U, Oswald E, Létisse F, Portais J-C. 2013. The carbon storage regulator (Csr) system exerts a nutrient-specific control over central metabolism in Escherichia coli strain Nissle 1917. PLoS One 8:e66386. doi:10.1371/journal.pone.0066386. 23840455 PMC3688793

[62] Iguchi A, Thomson NR, Ogura Y, Saunders D, Ooka T, Henderson IR, Harris D, Asadulghani M, Kurokawa K, Dean P, Kenny B, Quail MA, Thurston S, Dougan G, Hayashi T, Parkhill J, Frankel G. 2009. Complete genome sequence and comparative genome analysis of enteropathogenic Escherichia coli O127:H6 strain E2348/69. J Bacteriol 191:347–354. doi:10.1128/JB.01238-08. 18952797 PMC2612414

[63] Miquel S, Peyretaillade E, Claret L, de Vallée A, Dossat C, Vacherie B, Zineb EH, Segurens B, Barbe V, Sauvanet P, Neut C, Colombel J-F, Medigue C, Mojica FJM, Peyret P, Bonnet R, Darfeuille-Michaud A. 2010. Complete genome sequence of Crohn’s disease-associated adherent-invasive E. coli strain LF82. PLoS One 5:e12714. doi:10.1371/journal.pone.0012714. 20862302 PMC2941450

[64] Reuven NB, Deutscher MP. 1993. Multiple exoribonucleases are required for the 3’ processing of Escherichia coli tRNA precursors in vivo. FASEB J 7:143–148. doi:10.1096/fasebj.7.1.8422961. 8422961

[65] Pérès SY, Marchès O, Daigle F, Nougayrède JP, Herault F, Tasca C, De Rycke J, Oswald E. 1997. A new cytolethal distending toxin (CDT) from Escherichia coli producing CNF2 blocks HeLa cell division in G2/M phase. Mol Microbiol 24:1095–1107. doi:10.1046/j.1365-2958.1997.4181785.x. 9220015

[66] Morin M, Ropers D, Cinquemani E, Portais J-C, Enjalbert B, Cocaign-Bousquet M. 2017. The Csr system regulates *Escherichia coli* fitness by controlling glycogen accumulation and energy levels. mBio 8:e01628-17. doi:10.1128/mBio.01628-17. 29089432 PMC5666160

