# Supplementary material for: Availability of the Molecular Switch XylR Controls Phenotypic Heterogeneity and Lag Duration during Escherichia coli Adaptation from Glucose to Xylose
Source: mBio. 2020 Dec 22;11(6):e02938-20. doi: 10.1128/mBio.02938-20 (PMC8534289; doi:10.1128/mBio.02938-20)
Supplement: FIG S1 [file mbio.02938-20-sf001.pdf]

	Median signal intensity mRFP1 (u.a.f)	Median signal intensity mTagBFP (u.a.f)
Glucose medium	3.55	47.04
Xylose medium	120.00	44.47

Figure S1: Median fluorescence intensity of *E. coli* BW25113 strain carrying the dual fluorescence plasmid pSB1C3 P_{xylA}-mRFP1 P_{ihfBA}-mTagBFP in M9 glucose medium versus in M9 xylose medium. Fluorescence was measured by cytometry during the exponential growth phase. The Y2-A canal was used to quantify mRFP1 fluorescence intensity (inducible on xylose) and the V1-A canal to quantify mTagBFP fluorescence intensity (constitutive).
