# Supplementary material for: Availability of the Molecular Switch XylR Controls Phenotypic Heterogeneity and Lag Duration during Escherichia coli Adaptation from Glucose to Xylose
Source: mBio. 2020 Dec 22;11(6):e02938-20. doi: 10.1128/mBio.02938-20 (PMC8534289; doi:10.1128/mBio.02938-20)
Supplement: FIG S3 [file mbio.02938-20-sf003.pdf]

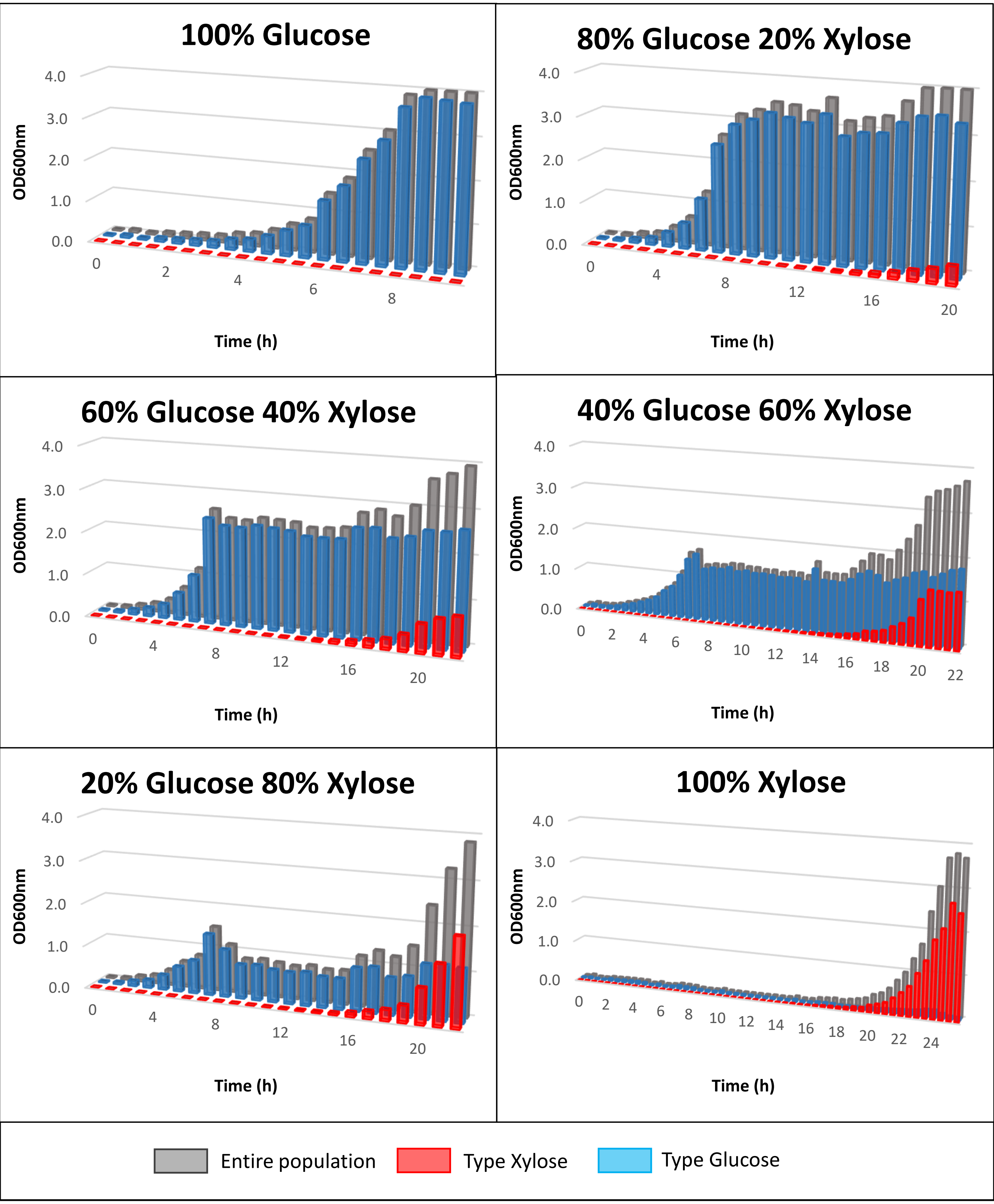

Figure S3: The biomass of each subpopulation extrapolated from the percentages resulting from the flow cytometry analyses during the growth on 100% glucose, 80% glucose and 20% xylose mix, 60% glucose and 40% xylose mix, 40% glucose and 60% xylose mix, 80% glucose and 20% xylose mix or 100% xylose (glucose type cells in blue, xylose type cells in red and the whole population in grey).
