# Supplementary material for: Availability of the Molecular Switch XylR Controls Phenotypic Heterogeneity and Lag Duration during Escherichia coli Adaptation from Glucose to Xylose
Source: mBio. 2020 Dec 22;11(6):e02938-20. doi: 10.1128/mBio.02938-20 (PMC8534289; doi:10.1128/mBio.02938-20)
Supplement: TEXT S1 [file mbio.02938-20-s0001.pdf]

Mathematical modeling of an *E. coli* batch culture on glucose and xylose

April 30, 2020

1 Stochastic model description

We are interested in a batch culture of an *E. coli* strain growing on glucose and xylose. We assume that for any individual, the consumption of xylose is initiated by the binding of the xylR protein to DNA. We thus split the whole population into the following classes.

- **Glucose consumers** with a basal level of xylR protein. We denote by $X_1(t)$ the number of such individuals at time t. We call this class "Class X_1".
- **Glucose consumers** with a high level of xylR protein. We denote by $X_2(t)$ their number at time t. This class contains individuals that have already grown on xylose and thus have an over-expressed xylR protein that can be progressively lost in divisions. We call this class "Class X_2".
- **Xylose consumers**, with always a high level of xylR protein. We denote by $Y(t)$ their number at time t. We call this class "Class Y".

The ressource concentrations are described by the process

$$S(t) = (S_1(t), S_2(t))$$

in which the first component represents glucose and the second one, xylose.

In order to investigate this, we introduce a Markovian model evolving according to the following three mechanisms.

1. **Cell division.** Each individual divides at rate $b_1(S_1(t))$ if it consumes glucose, and $b_2(S_2(t))$ if it consumes xylose. In addition, a glucose consumer with an over-expressed xylR level (Class X_2) gives birth to a cell with a basal xylR level (Class X_1) with probability $\alpha \in (0, 1)$ due to xylR dilution during cell division. This description corresponds to the following transitions.

   - Births in Class X_1:

   $$(X_1, X_2, Y) \longrightarrow (X_1 + 1, X_2, Y) \ \text{ at rate } \ b_1(S_1)X_1\,.$$

- Births in Class X_2, taking into account xylR dilution:

$$(X_1, X_2, Y) \longrightarrow (X_1 + 1, X_2, Y) \text{ at rate } \alpha b_1(S_1) X_2 \,,$$
$$(X_1, X_2, Y) \longrightarrow (X_1, X_2 + 1, Y) \text{ at rate } (1 - \alpha) b_1(S_1) X_2 \,.$$

- Births in Class Y:

$$(X_1, X_2, Y) \longrightarrow (X_1, X_2, Y + 1) \text{ at rate } b_2(S_2) Y \,.$$

In this model we consider that the division rates are of Monod type:

$$b_1(S_1) = \mu_1 \frac{S_1}{k_1 + S_1} \,, \quad b_2(S_2) = \mu_2 \frac{S_2}{k_2 + S_2} \,. \tag{1}$$

2. **State transitions.** For each glucose consumer, a copy of xylR protein must bind to DNA in order to initiate the switch toward xylose consumption. This event occurs at rate $\lambda(S)$ when the bacterium has a low level of xylR protein and at rate $(1 + \theta)\lambda(S)$ when it has a high level, in which $\theta > 0$ is an increase coefficient due to the abundance of xylR copies. This rives rise to the following transitions.

   - Transitions from Class X_1 to Class Y:

   $$(X_1, X_2, Y) \longrightarrow (X_1 - 1, X_2, Y + 1) \text{ at rate } \lambda(S) X_1 \,.$$

   - Transitions from Class X_2 to Class Y:

   $$(X_1, X_2, Y) \longrightarrow (X_1, X_2 - 1, Y + 1) \text{ at rate } (1 + \theta)\lambda(S) X_2 \,.$$

   The transition rate $\lambda(S)$ is inhibited by glucose, and is modeled using a Monod-type rate function and a classic inhibition function as

   $$\lambda(S) = \frac{\bar{\lambda} S_2}{k_f + S_2} \cdot \frac{k_i}{k_i + S_1} \,. \tag{2}$$

   Likewise, in presence of glucose, a xylose consumer switches toward glucose consumption at rate $\eta(S_1)$, giving rise to the transition

   $$(X_1, X_2, Y) \longrightarrow (X_1, X_2 + 1, Y - 1) \text{ at rate } \eta(S_1) Y \,,$$

   and we take

   $$\eta(S_1) = \frac{\bar{\eta} S_1}{k_d + S_1} \,.$$

3. **Resource dynamics.** It is driven by a continuous consumption of each sugar by cells in order to create biomass and divide. This is modeled for a batch of volume V by the equations

   $$\begin{cases} \dfrac{dS_1(t)}{dt} = -\dfrac{1}{Y_G V} b_1(S_1(t))(X_1(t) + X_2(t)) \,, \\ \dfrac{dS_2(t)}{dt} = -\dfrac{1}{Y_X V} b_2(S_2(t)) Y(t) \,, \end{cases} \tag{3}$$

   in which the yield coefficients Y_G and Y_X are expressed in terms of individuals per mass of glucose and xylose, respectively.

2 Large population approximation

2.1 Convergence of the stochastic model to a deterministic limit model

During experiments, broth contains more than a billion individuals. We thus need to rescale the population as well as the yield coefficients by considering that the population size at time 0 is of order of magnitude K, where $K > 0$ is large.

The subpopulation sizes and concentrations are now parameterized by K which can be seen as the carrying capacity of the system (in the ecological terminology). Moreover, we assume that each individual is weighted by $1/K$ so that the quantities

$$x_1^K(t) = \frac{X_1^K(t)}{VK}, \quad x_2^K(t) = \frac{X_2^K(t)}{VK}, \quad y^K(t) = \frac{Y^K(t)}{VK},$$

represent the mass densities of each class. We assume that the initial conditions are given in terms of $(x_1(0), x_2(0), y(0), s_1(0), s_2(0))$ not depending on K in the form

$$X_1^K(0) = \lfloor KVx_1(0) \rfloor, \quad X_2^K(0) = \lfloor KVx_2(0) \rfloor, \quad Y^K(0) = \lfloor KVy(0) \rfloor,$$

(where $\lfloor x \rfloor$ is the integer part of x) and

$$S_1^K(0) = s_1(0), \quad S_2^K(0) = s_2(0).$$

We also assume that the yield coefficients are proportional to K: for fixed y_G and y_X,

$$Y_G = y_G K, \quad Y_X = y_X K.$$

When K is very large (tends to infinity), one can prove that the stochastic process

$$(x_1^K(t), x_2^K(t), y^K(t), S_1^K(t), S_2^K(t))_{t \geq 0}$$

is well approximated by the differential system

$$\begin{cases} \dfrac{dx_1}{dt} = (b_1(s_1) - \lambda(s))\, x_1 + \alpha b_1(s_1) x_2, \\ \dfrac{dx_2}{dt} = ((1-\alpha) b_1(s_1) - (1+\theta)\lambda(s))\, x_2 + \eta(s_1) y, \\ \dfrac{dy}{dt} = (b_2(s_2) - \eta(s_1))\, y + \lambda(s)\, (x_1 + (1+\theta) x_2), \\ \dfrac{ds_1}{dt} = -\dfrac{1}{y_G} b_1(s_1)\, (x_1 + x_2), \\ \dfrac{ds_2}{dt} = -\dfrac{1}{y_X} b_2(s_2)\, y, \end{cases} \tag{4}$$

with initial condition $(x_1(0), x_2(0), y(0), s_1(0), s_2(0))$. The proof of this fact is based on classical arguments of tightness and identification (see Ethier and Kurtz (1986) for an abstract exposition, and Anderson and Kurtz (2015) and Bansaye and Méléard (2015) for a pedagogical treatment).

2.2 Identifiability of the model

Let us first recapitulate the different parameters involved in the system (4).

Parameter	Signification
μ_1	maximal growth rate for glucose consumers
k_1	Michaelis–Menten coefficient for glucose
μ_2	maximal growth rate for xylose consumers
k_2	Michaelis–Menten coefficient for xylose
$\bar{\lambda}$	maximal transition rate from glucose to xylose consumption
k_f	regulating coefficient for xylR protein activation and binding
k_i	inhibition coefficient of switches from glucose to xylose consumption
$\bar{\eta}$	maximal transition rate from xylose to glucose consumption
k_d	regulating coefficient of switches from glucose to xylose consumption
α	xylR protein dilution coefficient per cellular division
θ	xylR fixation's amplification coefficient
y_G	yield coefficient for glucose consumers
y_X	yield coefficient for xylose consumers

Table 1: Parameters of the differential system.

Roughly speaking, the identifiability of a model is defined as the ability to identify a unique set of its parameters from the available data (see Walter and Pronzato (1997)). Since we are not able to distinguish experimentally the sub-populations x_1 and x_2 in the model (4), we can show that this model is not actually identifiable from the available data. In a general situation, as a first attempt and although it cannot be presented as a rigorous proof, a practical way to test identifiability is to carry out parametric identification from varied initial conditions. If the optimization algorithm always converges towards approximately the same set of parameters, then we may have some faith in this estimation. If the optimization algorithm fails do do so, then it is likely that the model is not identifiable. To overcome this last difficulty, we can try to obtain new data, fix certain parameters of which the values can be found in the literature or obtained from experts, or even modify the structure or the dimension of the model. In order to solve our problem, we have adopted the last solution, as will be described hereafter.

For the data collected during the experiments, the whole initial population is composed of glucose consumers with a basal level of xylR protein (hence, $x_2(0) = y(0) = 0$). Since the transition rate from glucose to xylose consumption is very small when glucose is abundant (see xylose variations in data), one can conclude that the class of glucose consumers with high level of xylR protein is insignificant at the observation size scale.

Hence, we propose in this case the following approximation of model (4):

$$\begin{cases} \dfrac{d\tilde{x}}{dt} = \left[b_1(\tilde{s}_1) - \lambda(\tilde{s})\right]\tilde{x}\,, \\ \dfrac{d\tilde{y}}{dt} = b_2(\tilde{s}_2)\tilde{y} + \lambda(\tilde{s})\tilde{x}\,, \\ \dfrac{d\tilde{s}_1}{dt} = -\dfrac{1}{y_G} b_1\left(\tilde{s}_1\right)\tilde{x}\,, \\ \dfrac{d\tilde{s}_2}{dt} = -\dfrac{1}{y_X} b_2\left(\tilde{s}_2\right)\tilde{y}\,, \end{cases} \tag{5}$$

where $(\tilde{x}_t)_{t\geq 0}$ represents the global subpopulation of glucose consumers, b_1 and b_2 are defined in (1), and λ is defined in (2).

Remark. *This model reduction imposes the following limitation. Since the compartment x_2 of model (4) has been omitted in model (5), the latter will not be able to predict the dynamics of experiments for which the dilution of xylR may play an important role, in which cells starting in the y compartment may switch to the x_2 compartment to grow on glucose while still having a high level of xylR.*

2.3 Parameter estimation

We use a least-square method to estimate the parameters

$$\Theta = (\mu_1, k_1, \mu_2, k_2, \bar{\lambda}, k_f, k_i, \kappa_1, \kappa_2)$$

by minimizing the distance between the model (5) and the data by implementing the tool **CMA_Evolution_Strategy** developed on **Python** by the INRIA team RandOpt (see Hansen and Ostermeier (2001)). We will denote by $(\bar{x}, \bar{y}, \bar{s})$ the experimental data.

1. **The strain with the plasmid**. We use the cost function given by

$$\mathcal{J}_1(\Theta) = \sum_{i=1}^{N_1} \sum_{t\in\mathbb{T}_i^{(1)}} \left\{ c_1 \left(\tilde{x}^{i,\Theta}(t) - \bar{x}^i(t)\right)^2 + c_2 \left(\tilde{y}^{i,\Theta}(t) - \bar{y}^i(t)\right)^2 \right.$$
$$\left. + c_3 \left(\tilde{s}_1^{i,\Theta}(t) - \bar{s}_1^i(t)\right)^2 + c_4 \left(\tilde{s}_2^{i,\Theta}(t) - \bar{s}_2^i(t)\right)^2 \right\}.$$

2. **The wild type**. In this case, the subpopulations are indistinguishable. The cost function will then be given by

$$\mathcal{J}_2(\Theta) = \sum_{i=1}^{N_2} \sum_{t\in\mathbb{T}_i^{(2)}} \left\{ c_1' \left(\tilde{x}^{i,\Theta}(t) + \tilde{y}^{i,\Theta}(t) - \bar{x}^i(t) - \bar{y}^i(t)\right)^2 \right.$$
$$\left. + c_2' \left(\tilde{s}_1^{i,\Theta}(t) - \bar{s}_1^i(t)\right)^2 + c_3' \left(\tilde{s}_2^{i,\Theta}(t) - \bar{s}_2^i(t)\right)^2 \right\}.$$

For $j = 1, 2$ indexing the strain type, N_j is the number of experiments and $\mathbb{T}_i^{(j)}$ the time mesh for the i-th experiment. We assume that both strains share the same values of some parameters, and that the other parameters are influenced by the plasmid. The corresponding mean values of the estimations are given in the following table.

Parameter	Estimation (with plasmid)	Estimation (empty plasmid)	Unit
μ_1	**6.50e-01**	**7.68e-01**	h^{-1}
k_1	5.70e-03	5.70e-03	g/L
μ_2	**5.41e-01**	**5.87e-01**	h^{-1}
k_2	1.33e-01	1.33e-01	g/L
$\bar{\lambda}$	**2.02e-04**	**2.09e+00**	h^{-1}
k_f	**6.71e-01**	**1.59e-08**	g/L
k_i	**4.98e+00**	**8.57e-16**	g/L
y_G	5.70e-01	5.70e-01	$g_{biomass}/g_{glucose}$
y_X	**4.00e-01**	**4.50e-01**	$g_{biomass}/g_{xylose}$

Table 2: Parameter estimation. Parameters under plasmid influence appear in bold.

These values reveal that the strain with plasmid grows slower than the one with an empty plasmid, whatever the substrat. In addition, their inability to switch immediately toward xylose consumption is strongly due to the plasmid that traps xylR copies (for the strain with plasmid) and the catabolic repression which induces an important inhibition of glucose on this transition (for the strain with an empty plasmid). In this last case, the switching rate is very high after glucose exhaustion. Many individuals are thus able to switch per time unit and hence the lag phase is very short.

Specifically, after glucose exhaustion at time t_0 a proportion

$$p(t) = 1 - \exp\left(-\bar{\lambda}\int_{t_0}^{t} \frac{s_2(u)}{k_f + s_2(u)}\,du\right) \tag{6}$$

of the resident population that had grown on glucose will have switched before time $t \geq t_0$. Hence, on a high xylose medium, the proportion $1 - e^{-\bar{\lambda}}$ of the resident population switches toward xylose consumption per hour:

Strain	$1 - e^{-\bar{\lambda}}$	Observation
With plasmid	2.02e-04	In this case $p(t) \leq 1 - e^{-\bar{\lambda}(t-t_0)}$ and then less than 2 individuals over 10.000 can switch per hour
Empty plasmid	8.76e-01	In this case $p(t) \approx 1 - e^{-\bar{\lambda}(t-t_0)}$ and then about 8760 individuals over 10.000 can switch per hour

Table 3: Proportion of individuals that switch per hour.

References

Anderson, D. F. and Kurtz, T. G. (2015). *Stochastic Analysis of Biochemical Systems*. Springer.

Bansaye, V. and Méléard, S. (2015). *Stochastic Models for Structured Populations: Scaling Limits and Long Time Behavior*. Springer.

Ethier, S. N. and Kurtz, T. G. (1986). *Markov Processes: Characterization and Convergence*. John Wiley & Sons, Inc.

Hansen, N. and Ostermeier, A. (2001). Completely derandomized self-adaptation in evolution strategies. *Evolutionary Computation*, 9(2):159–195.

Walter, E. and Pronzato, L. (1997). *Identification of Parametric Models from Experimental Data*. Springer.
