# Supplementary material for: Availability of the Molecular Switch XylR Controls Phenotypic Heterogeneity and Lag Duration during Escherichia coli Adaptation from Glucose to Xylose
Source: mBio. 2020 Dec 22;11(6):e02938-20. doi: 10.1128/mBio.02938-20 (PMC8534289; doi:10.1128/mBio.02938-20)
Supplement: FIG S4 [file mbio.02938-20-sf004.pdf]

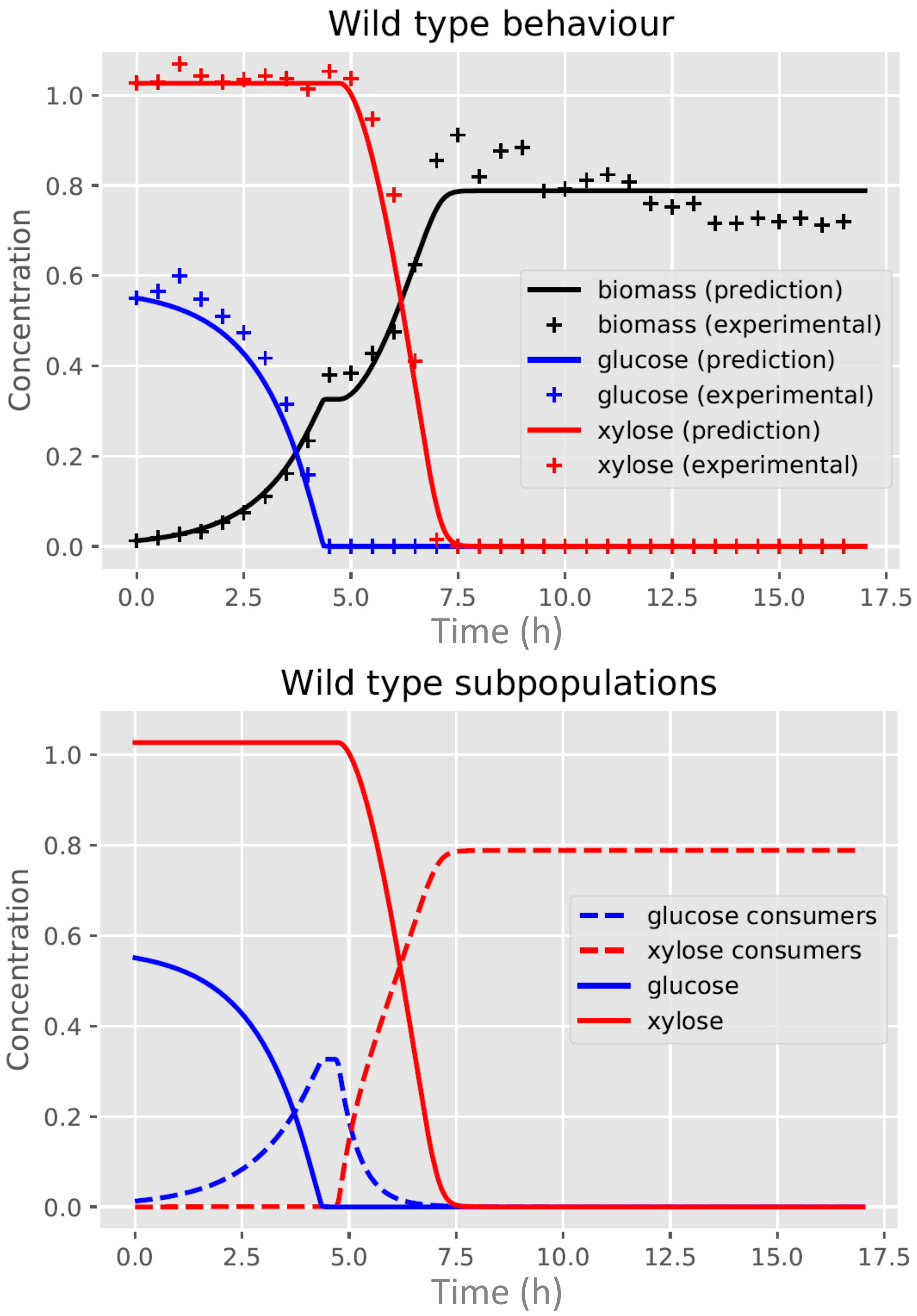

Figure S4: Behavior of wild type strain BW25113 on a glucose-xylose mix. Experimental and prediction data are presented for biomass concentration ($g.L^{-1}$) and substrates ($g.L^{-1}$) in the top row and prediction data for subpopulations in the bottom row of graphs.
