# Supplementary material for: Availability of the Molecular Switch XylR Controls Phenotypic Heterogeneity and Lag Duration during Escherichia coli Adaptation from Glucose to Xylose
Source: mBio. 2020 Dec 22;11(6):e02938-20. doi: 10.1128/mBio.02938-20 (PMC8534289; doi:10.1128/mBio.02938-20)
Supplement: FIG S5 [file mbio.02938-20-sf005.pdf]

CGCGGCCGCTTCTAGAATTTTTAGCAACTAAACAGGGGAAAACTAATACAGAAATTTATCTTTCG
ATTACGATATATGGTTTATTTCTTGATTTATGACCGAGATCTTACTTTTGTTGCGCAATTGTACTTAT
TGCATTTTTCTCTTCGAGGAATTACCCAGTTTCATCATTCCATTTTATTTTGCGAGCGAGCGCACAC
TTGTGTAAATTCTCAATAGCAGTGTGTATTAACATTAATGAGCAACTGAAAGGGAGTGCCCAATAT
TACGACATCATCCATCACCCGCGGCATTACCTGATTATGGAGTTCAATA

Figure S5: Sequence of the xylA mutated promoter used in this study. The xylR fixation sites are in red and mutation in these sites are in green.
