# Supplementary material for: Availability of the Molecular Switch XylR Controls Phenotypic Heterogeneity and Lag Duration during Escherichia coli Adaptation from Glucose to Xylose
Source: mBio. 2020 Dec 22;11(6):e02938-20. doi: 10.1128/mBio.02938-20 (PMC8534289; doi:10.1128/mBio.02938-20)
Supplement: FIG S6 [file mbio.02938-20-sf006.pdf]

Name	Sequence	Utilisation
REITZER_xylA3	ATGTACTAGTATTGAACTCCATAATCAGG	xylA promoter amplification
REITZER_xylA5	ATGTGAATTCGCGGCCGCTTCTAGAATTTTTAGCAACTAAAC	xylA promoter amplification
New-ihfB3	ATGTACTAGTGATTCCTCCGGTTCCTTAAAGG	ihfB promoter amplification
ihfB5-800	ATGTGAATTCGCGGCCGCTTCTAGAAGTTGACGCTAAAGGCGCAAC	ihfB promoter amplification
cysG3-800	ATGTACTAGTGTAAAAACCCCTTAGTAATTAACC	cysG promoter amplification
cysG5-801	ATGTGAATTCGCGGCCGCTTCTAGACTATTAATAAGTGTGCGGCTAAC	cysG promoter amplification
gib3_xylR_for	TTACACACTAGAATGTTTACTAAACGTCACCGCATC	xylR amplification
gib3_xylR_rev	TTCTAGTGTCTACAACATGACCTCGCTATTTACATC	xylR amplification
XylR_CysG_for	CATCTACCTGCCTGGACAGCACTATTAATAAGTGTGC	pMET219-XylR construction
XylR_CysG_rev	TCACTATGGCGTGCTGCTAGTGTCTACAACATGACCTC	pMET219-XylR construction
pBR322_rev_gib2	TGCTGTCCAGGCAGGTAGATGAC	pMET219-XylR construction
pBR322_for_gib2	CTAGCAGCACGCCATAGTGACTG	pMET219-XylR construction

Figure S6: List of primers used to build the plasmids used in this study.
