# Supplementary material for: Availability of the Molecular Switch XylR Controls Phenotypic Heterogeneity and Lag Duration during Escherichia coli Adaptation from Glucose to Xylose
Source: mBio. 2020 Dec 22;11(6):e02938-20. doi: 10.1128/mBio.02938-20 (PMC8534289; doi:10.1128/mBio.02938-20)
Supplement: FIG S7 [file mbio.02938-20-sf007.pdf]

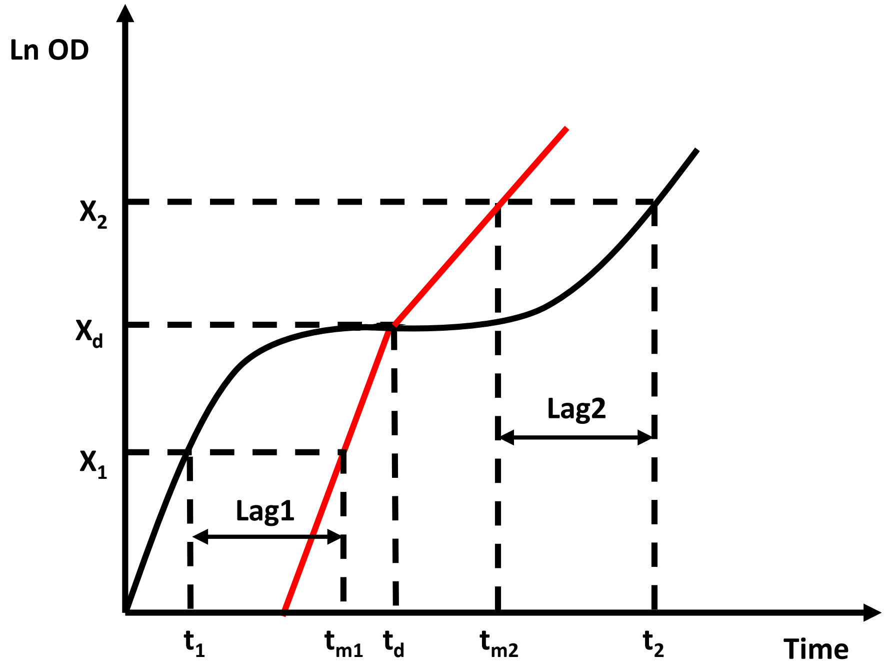

Figure S7: Demonstration of the diauxic lag formula

Here we consider the diauxic lagc as the time lost in comparison to hypothetical growth where a first exponential phase is immediately followed by a second exponential phase (in red in the figure).

Xd: a biomass point reached during the diauxic lag.

µmax1: the maximal growth rate on the first substrate.

µmax2: the maximal growth rate on the second substrate.

X1: biomass reached during the exponential growth on the first substrate at time *t1 or at time tm1* if the growth rate was theoretically maximal until *td*

X2: biomass reached during the exponential growth on the second substrate at time *t2* or at time *tm2* if the growth rate was theoretically maximal from *td.*

The diauxic lag is thus equal to lag1 (*tm1-t1*) for the glucose growth phase plus lag2 (*t2 - tm2*) for the xylose growth phase. Based on the formula proposed by Enjalbert (Enjalbert et al., 2015), we demonstrated the diauxic lag calculation as followed:

- Demonstration for lag1

µmax1 = ln(Xd/X1)/(td-tm1), thus *(td-tm1) = ln(Xd/X1)/µmax1*

Given that Lag1 = *(tm1-t1) = td-(td-tm1)-t1*

Thus *(tm1-t1) = td-(ln(Xd/X1)/µmax1) - t 1*

- Demonstration for lag2

µmax2 = ln(X2/Xd)/(tm2-td), thus *(tm2-td) = ln(X2/Xd)/µmax2*

Given that lag2 = *(t2-tm2) = (t2-td)-(tm2-td)*

Thus *(t2-tm2) = (t2-td)-(ln(X2/Xd)/µmax2)*

Since diauxie lag = lag1+lag2

Diauxic lag = *td-(ln(Xd/X1)/µmax1)-t1+(t2-td)-(ln(X2/Xd)/µmax2)*

So that Diauxic lag = *t2-t1-(ln(Xd/X1)/µmax1)-(ln(X2/Xd)/µmax2)*
